# All‐Trans Retinoic Acid Promotes a Tumor Suppressive OTUD6B‐*β*‐TrCP‐SNAIL Axis in Esophageal Squamous Cell Carcinoma and Enhances Immunotherapy

**DOI:** 10.1002/advs.202207458

**Published:** 2023-04-10

**Authors:** Lei Li, Rui Zhu, Honghong Zhou, Chun‐Ping Cui, Xiao Yu, Yuhao Liu, Yin Yin, Yang Li, Riyue Feng, Jonathan P. Katz, Yahui Zhao, Yun Zhang, Lingqiang Zhang, Zhihua Liu

**Affiliations:** ^1^ State Key Laboratory of Molecular Oncology National Cancer Center/National Clinical Research Center for Cancer/Cancer Hospital Chinese Academy of Medical Sciences and Peking Union Medical College Beijing 100021 P. R. China; ^2^ Department of Radiation Oncology National Cancer Center/National Clinical Research Center for Cancer/Cancer Hospital & Shenzhen Hospital Chinese Academy of Medical Sciences and Peking Union Medical College Shenzhen 518116 P. R. China; ^3^ State Key Laboratory of Proteomics National Center for Protein Sciences (Beijing) Beijing Institute of Lifeomics Beijing 100850 P. R. China; ^4^ Gastroenterology Division Department of Medicine University of Pennsylvania Philadelphia PA 19104 USA

**Keywords:** all‐trans retinoic acid, anti‐PD‐1, deubiquitination, metastasis, tumorigenesis, tumor‐initiating cell property

## Abstract

*β*‐TrCP is an E3 ubiquitin ligase that plays important roles in multiple human cancers including esophageal squamous cell carcinoma (ESCC). Analysis of ESCC patient samples reveal that only protein level but not transcript level of *β*‐TrCP associated with patient prognosis, suggesting regulators of *β*‐TrCP protein stability play an essential role in ESCC progression and may be novel targets to develop ESCC therapies. Although *β*‐TrCP stability is known to be mediated by the ubiquitin‐proteasome system, it is unclear which enzymes play a major role to determine *β*‐TrCP stability in the context of ESCC. In this study, OTUD6B is identified as a potent deubiquitinase of *β*‐TrCP that suppress ESCC progression through the OTUD6B‐*β*‐TrCP‐SNAIL axis. Low OTUD6B expression is associated with a poor prognosis of ESCC patients. Importantly, all‐trans retinoic acid (ATRA) is found to promote OTUD6B translation and thus suppress ESCC tumor growth and enhance the response of ESCC tumors to anti‐PD‐1 immunotherapies. These findings demonstrate that OTUD6B is a crucial deubiquitinase of *β*‐TrCP in ESCC and suggest combination of ATRA and anti‐PD‐1 immune checkpoint inhibitor may benefit a cohort of ESCC patients.

## Graphical abstract

This work identifies OTUD6B as a potent deubiquitinase of *β*‐TrCP that suppresses esophageal squamous cell carcinoma (ESCC) progression through the OTUD6B‐*β*‐TrCP‐SNAIL axis. Low OTUD6B expression is associated with a poor prognosis of ESCC patients. Importantly, all‐trans retinoic acid is found to promote OTUD6B translation and thus suppresses ESCC tumor growth and enhances the response of ESCC tumors to anti‐PD‐1 immunotherapies.

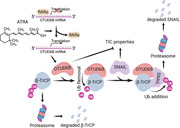

## 1 Introduction

Esophageal carcinoma is the seventh most prevalent cancer and the sixth leading cause of cancer‐related mortality in the world, causing 544 076 deaths in 2020.^[^ ^1^ ^]^ Esophageal squamous cell carcinoma (ESCC) is the most common subtype of esophageal carcinoma, accounting for ≈90% of all esophageal cancer patients.^[^ ^2^ ^]^ Various therapies are used in ESCC patients, such as surgery, chemotherapy, and radiotherapy, but the 5‐year survival rate remains only ≈30%.^[^ ^3^ ^]^ Accordingly, it is necessary to investigate the concrete mechanism of ESCC tumorigenesis and develop more effective therapies.

F‐box proteins are proteins containing at least one F‐box domain, which are commonly misregulated in cancer.^[^ ^4^ ^]^ One of the well‐characterized F‐box proteins, *β*‐TrCP, has been shown to play an important role in regulating tumor development and metastasis for multiple cancer types including esophageal carcinoma.^[^ ^5^ ^]^ As an E3 ubiquitin ligase, *β*‐TrCP is involved in the ubiquitination of some well‐characterized substrates that regulate cell growth, apoptosis and stemness, including SOX9, SNAIL, TAZ, and YAP1.^[^ ^6^ ^]^ It has been shown that in esophageal adenocarcinomas, down‐regulation of *β*‐TrCP promotes YAP1 protein stability and thus facilitates tumorigenesis.^[^ ^7^ ^]^ In ESCC, tribbles pseudokinase 3 inhibits *β*‐TrCP‐mediated TAZ ubiquitination and degradation to promote tumor‐initiating cell (TIC) properties and radioresistance of cancer cells.^[^ ^5d^ ^]^ In addition, it has been reported that a significant percentage of ESCC patient specimens shows reduced *β*‐TrCP expression.^[^ ^8^ ^]^

*β*‐TrCP is involved in the process of protein ubiquitination and its own stability is heavily regulated by this process.^[^ ^9^ ^]^ For example, ubiquitin ligases SUMRF2 and Skp2 have been found to promote the ubiquitination of *β*‐TrCP and in turn induce its degradation.^[^ ^10^ ^]^ On the other hand, although ubiquitination is known as a reversible process and deubiquitinases (DUBs) can counterbalance the ubiquitination process,^[^ ^11^ ^]^ it is unclear whether certain deubiquitinating enzymes could reverse *β*‐TrCP ubiquitination. Given the importance of *β*‐TrCP in ESCC development and the potential to develop novel therapies through regulating *β*‐TrCP stability, it is in urgent need to investigate the process of *β*‐TrCP deubiquitination.

In this study, we identified OTUD6B as a potent DUB for *β*‐TrCP which plays a major role in regulating its stabilization. By increasing *β*‐TrCP protein level and activating an OTUD6B‐*β*‐TrCP‐SNAIL axis, OTUD6B suppresses the TIC properties of ESCC cells. In ESCC tumor samples, OTUD6B expression is positively correlated with *β*‐TrCP and its lower expression associates with a poor prognosis. Importantly, all‐trans retinoic acid (ATRA) was found to promote the OTUD6B translation, suppress the tumorigenesis and metastasis of ESCC, and enhance the response of established ESCC tumors to anti‐PD‐1 immunotherapy. These data suggest that ATRA could be applied to treat a cohort of OTUD6B‐expressing ESCC patients.

## 2 Results

### 2.1 OTUD6B Enhances *β*‐TrCP Protein Stability

**Figure 1:**
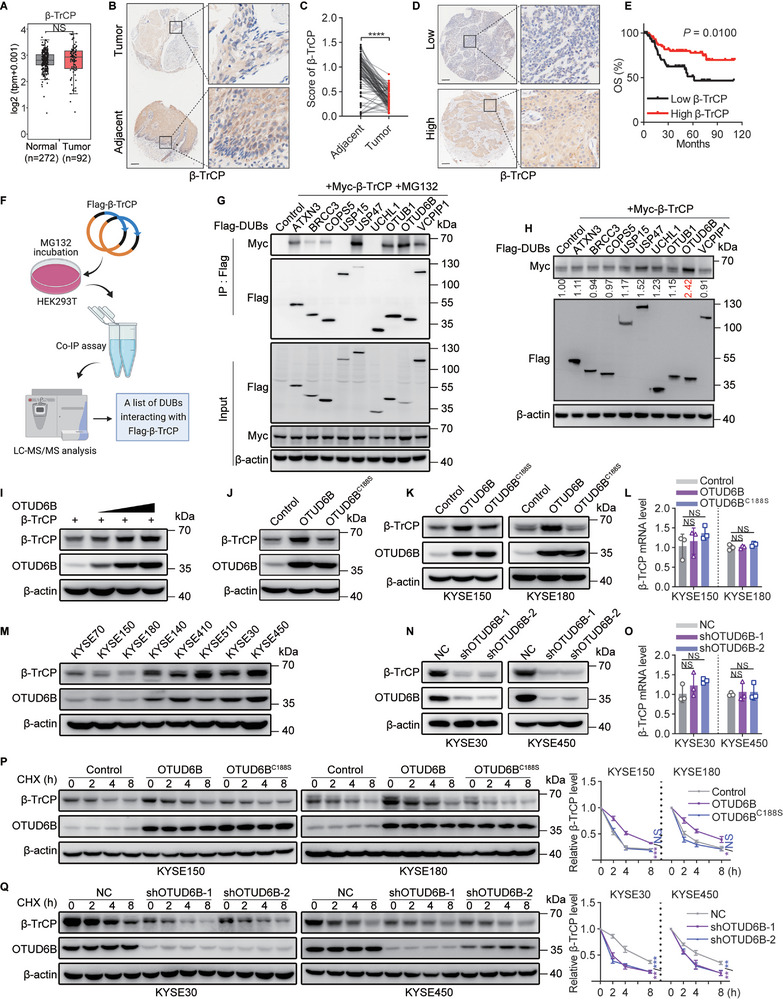
OTUD6B enhances *β*‐TrCP protein stability. A) The relative transcript levels of *β*‐TrCP in ESCC and normal tissues were analyzed using the transcriptome profiles in TCGA and GTEx data. Wilcoxon test, NS, not significant (*p* > 0.05). B) The expression of *β*‐TrCP in 85 paired ESCC and adjacent normal tissues was detected by immunohistochemistry, and C) the relative expression of *β*‐TrCP was analyzed. Paired *t*‐test, **** *p* < 0.0001. D) The expression of *β*‐TrCP in 128 ESCC tissues was detected by immunohistochemistry, and E) the overall survival of 128 ESCC patients stratified by OTUD6B expression. Two‐sided log‐rank test. F) A schematic diagram shows the immunoprecipitated screening for potential DUBs interacting with *β*‐TrCP. G) The indicated DUBs and *β*‐TrCP were co‐transfected into HEK293T cells. The interaction of potential DUBs and *β*‐TrCP was detected by immunoprecipitation. H) The indicated DUBs and *β*‐TrCP were co‐transfected into HEK293T cells. The expression of *β*‐TrCP was detected by immunoblotting and qualified. I) Increasing quantities of OTUD6B plasmid were transfected into HEK293T cells and *β*‐TrCP expression was detected. J) OTUD6B or OTUD6B^C188S^ was transfected into HEK293T cells, and *β*‐TrCP expression was detected. K) The protein and L) mRNA levels of *β*‐TrCP were detected in KYSE150 and KYSE180 cells overexpressing OTUD6B or OTUD6B^C188S^. *n* = 3, mean ± SEM, unpaired *t*‐test, NS, not significant (*p* > 0.05). M) The protein levels of OTUD6B and *β*‐TrCP in ESCC cell lines were detected. N) The protein and O) mRNA levels of *β*‐TrCP were detected in OTUD6B‐knockdown KYSE30 and KYSE450 cells. *n* = 3, mean ± SEM, unpaired *t*‐test, NS, not significant (*p* > 0.05). P) The degradation of *β*‐TrCP in OTUD6B or OTUD6B^C188S^ ‐overexpressing KYSE150 and KYSE180 cells treated with CHX (50 µg mL^−1^) for 2, 4, 8 h. *n* = 3, mean ± SD, unpaired *t*‐test, * *p* < 0.05, *** *p* < 0.001, NS, not significant (*p* > 0.05). Q) The degradation of *β*‐TrCP in OTUD6B‐knockdown KYSE30 and KYSE450 cells treated with CHX (50 µg mL^−1^) for 2, 4, 8 h. *n* = 3, mean ± SD, unpaired *t*‐test, ** *p* < 0.01, *** *p* < 0.001. The data shown in G‐K, M, N, P, and Q are from a representative experiment in at least two replicates.

To better understand the role of *β*‐TrCP in ESCC development, we first examined its mRNA and protein expression levels in published datasets and a cohort of ESCC patient samples we collected. Interestingly, we found that the transcript level of *β*‐TrCP in the ESCC tissues did not show a significant difference compared with normal tissues (**Figure** **1**A). However, at the protein level, *β*‐TrCP expressed at lower level in ESCC tissues than in adjacent normal tissues (Figure 1B,C), which is consistent with a previous report.^[^ ^8^ ^]^ The clinical proteomic tumor analysis consortium (CPTAC) data also suggest that decreased protein level of *β*‐TrCP exists in other cancer types, including breast invasive carcinoma, colon adenocarcinoma, and glioblastoma (GBM) (Figure S1A, Supporting Information). Importantly, a lower *β*‐TrCP protein level was associated with poor overall survival in ESCC patients (Figure 1D,E), suggesting its potential role as a tumor suppressor in this cancer type.

The observation that only *β*‐TrCP protein level, but not transcript level, correlates with ESCC prognosis suggests that the regulation of protein production and/or stability of *β*‐TrCP might play an essential role in regulating ESCC development. Since *β*‐TrCP is known to be heavily regulated by the ubiquitin‐proteasome system (UPS)^[^ ^10^ ^]^ and it remains unclear whether there are DUBs directly regulating *β*‐TrCP protein stability, we determined to focus on identifying DUPs for *β*‐TrCP and examining their functions in ESCC progression.

To do so, we performed an immunoprecipitation assay followed by mass spectrometry (MS) to screen potential DUBs interacting with *β*‐TrCP (Figure 1F and Figure S1B, Supporting Information). Through this method, we identified nine DUBs as potential regulators of *β*‐TrCP protein stability (Figure S1C, Supporting Information). Among the results we also found several proteins previously known to bind *β*‐TrCP, validating the accuracy of our screening (Figure S1D, Supporting Information). We then confirmed that 7 of these nine DUBs interacted with *β*‐TrCP, including ATXN3, BRCC3, COPS5, USP47, OTUB1, OTUD6B, and VCPIP1 (Figure 1G). To identify which DUBs functionally regulate *β*‐TrCP stability, we co‐transfected *β*‐TrCP and each of these potential DUBs into HEK293T cells. As shown in Figure 1H, OTUD6B was the most potent DUB to increase *β*‐TrCP protein level. Such effect was markedly stronger than USP47, which was recently shown to upregulate *β*‐TrCP protein level though it is unclear whether this regulation relies on its deubiquitinating activity.^[^ ^12^ ^]^ In addition, *β*‐TrCP protein level was increased by OTUD6B in a dose‐dependent manner (Figure 1I).

We then transfected the OTUD6B plasmid alone into HEK293T cells and found that ectopic expression of wild‐type OTUD6B, but not the catalytically inactive mutant OTUD6B^C188S^, increased endogenous *β*‐TrCP protein level (Figure 1J). We confirmed that in two ESCC cell lines, KYSE150 and KYSE180 cells, *β*‐TrCP protein level was also significantly increased by OTUD6B ectopic expression but *β*‐TrCP mRNA level was not influenced (Figure 1K,L). In line with this, endogenous OTUD6B and *β*‐TrCP protein levels showed a strong positive correlation among ESCC cell lines (Figure 1M). Consistently, knockdown of OTUD6B using short hairpin RNAs (shRNAs) resulted in a significant decrease in *β*‐TrCP protein but not *β*‐TrCP mRNA in KYSE30 and KYSE450 cells, two ESCC cell lines showing high endogenous OTUD6B expression level (Figure 1N,O). Moreover, only OTUD6B but no other OTU family members was able to regulate *β*‐TrCP, since knock down OTUD6A or OTUD2 did not increase *β*‐TrCP protein level in ESCC cells (Figure S1E–H, Supporting Information).

A cycloheximide (CHX) pulse‐chase experiment was then performed to confirm that OTUD6B stabilizes the *β*‐TrCP protein. To do so, *β*‐TrCP with wild‐type OTUD6B or OTUD6B^C188S^ were co‐transfected into HEK293T cells, and the stability of *β*‐TrCP was found to be prolonged in the presence of wild‐type OTUD6B but not OTUD6B^C188S^ mutant (Figure S1I, Supporting Information). The effect of OTUD6B on increasing the stability of *β*‐TrCP was significantly stronger than that of USP47 (Figure S1J, Supporting Information). Moreover, ectopic expression of OTUD6B increased the stability of endogenous *β*‐TrCP in KYSE150 and KYSE180 cells (Figure 1P). Conversely, *β*‐TrCP was degraded rapidly in OTUD6B‐knockdown KYSE30 and KYSE450 cells, whereas such effect was not observed in OTUD6A‐ or OTUD2‐knockdown cells (Figure 1Q and Figure S1K,L, Supporting Information). These results indicate that OTUD6B is a potent regulator of *β*‐TrCP protein stability.

### 2.2 OTUD6B Directly Interacts with and Deubiquitinates *β*‐TrCP

**Figure 2:**
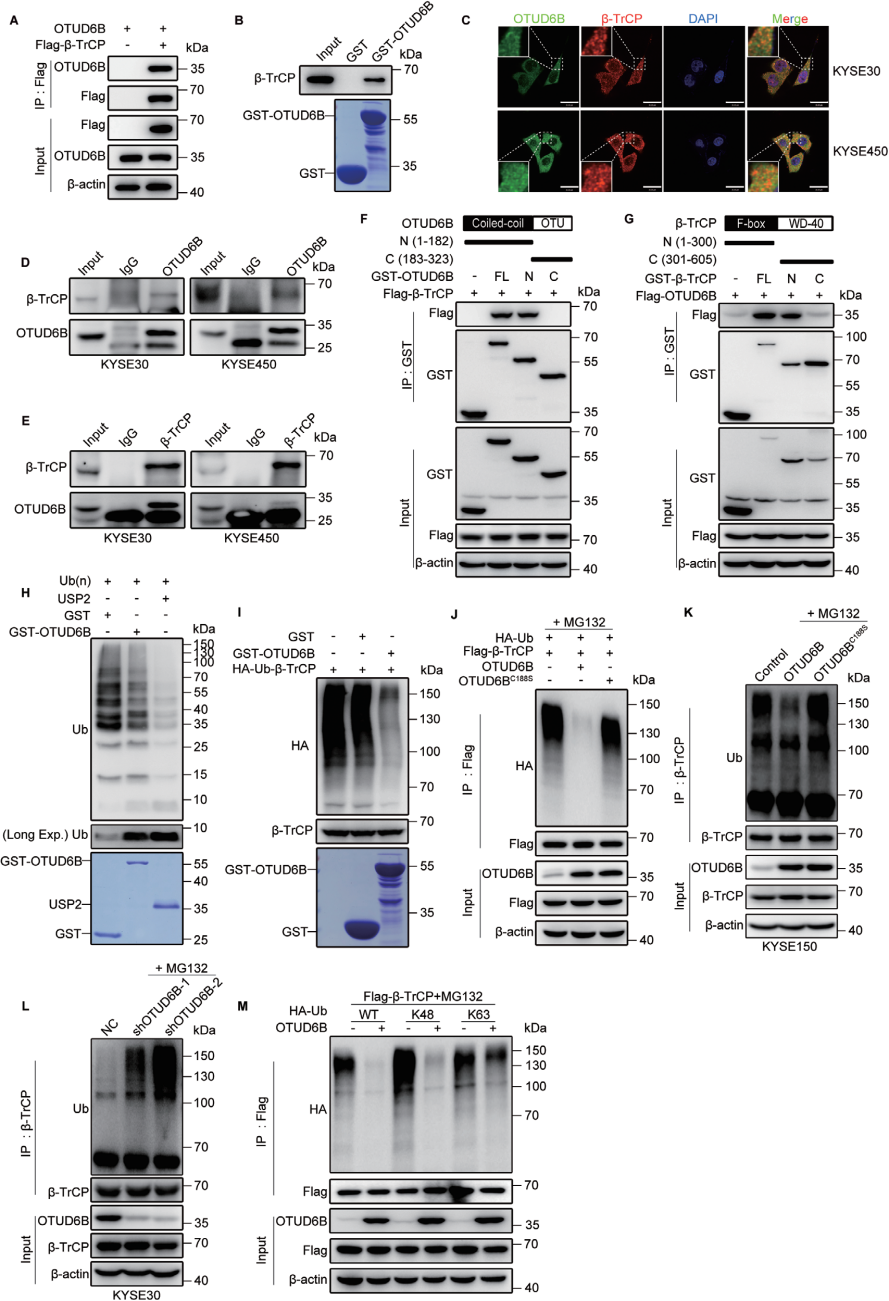
OTUD6B directly interacts with and deubiquitinates *β*‐TrCP. A) OTUD6B plasmid was co‐transfected with Flag‐*β*‐TrCP or vector into HEK293T cells. The interaction of *β*‐TrCP and OTUD6B was detected by immunoprecipitation assay. B) The GST pull‐down assay detected the interaction of *β*‐TrCP and OTUD6B in vitro. C) Endogenous OTUD6B (green) and *β*‐TrCP (red) in KYSE30 and KYSE450 cells were examined by immunofluorescence. Nuclei (blue) were stained with 4,6‐diamidino‐2‐phenylindole (DAPI). Scale bars, 30 µm. D) The interaction of *β*‐TrCP and OTUD6B was detected by immunoprecipitation assay using anti‐OTUD6B antibody or E) anti‐*β*‐TrCP antibody. F) A schematic diagram shows the structure of OTUD6B and its truncations. The interaction of *β*‐TrCP and OTUD6B truncations was detected by immunoprecipitation assay. G) A schematic diagram shows the structure of *β*‐TrCP and its truncations. The interaction of OTUD6B and *β*‐TrCP truncations was detected by immunoprecipitation assay. H) The Ub(n)‐ubiquitinylated substrate was incubated with purified GST, GST‐OTUD6B, or USP2 for 2 h at 37 °C. USP2 was used as a positive control. The status of ubiquitin was analyzed by immunoblotting. I) The purified ubiquitylated Flag‐*β*‐TrCP from HEK293T cells was incubated with purified GST‐OTUD6B or GST for 2 h at 37 °C. The ubiquitination status of *β*‐TrCP was analyzed by immunoblotting. J) Flag‐*β*‐TrCP and HA‐Ub were co‐expressed with OTUD6B or OTUD6B^C188S^ in HEK293T cells. The ubiquitination level of *β*‐TrCP was detected using an anti‐HA antibody. K) The ubiquitination level of *β*‐TrCP in OTUD6B‐ or OTUD6B^C188S^‐ overexpressing KYSE150 cells and L) OTUD6B‐knockdown KYSE30 cells. M) HA‐WT, K48, or K63 Ub (K48 and K63, all lysines of ubiquitin except for K48 and K63 were mutated to arginines) were co‐transfected with Flag‐*β*‐TrCP and OTUD6B into HEK293T cells. The ubiquitination level of *β*‐TrCP was detected with an anti‐HA antibody. All data from a representative experiment in at least two replicates.

As a DUB, OTUD6B is supposed to directly interact with *β*‐TrCP to govern its stability. To test this hypothesis, we co‐transfected Flag‐*β*‐TrCP and OTUD6B into HEK293T cells and then performed coimmunoprecipitation assays, as well as an GST‐pulldown assay by recombinant OTUD6B in vitro. As expected, OTUD6B was coimmunoprecipitated with *β*‐TrCP both in vivo and in vitro, demonstrating their direct interaction (**Figure** **2**A,B and Figure S2A, Supporting Information). This is further confirmed by their colocalization in ESCC cells as examined by confocal microscopy (Figure 2C). Moreover, similar to HEK293T cells, in KYSE30 and KYSE450 cells, OTUD6B and *β*‐TrCP were coimmunoprecipitated, indicating their interaction in ESCC cells (Figure 2D,E).

To identify the regions of OTUD6B responsible for the interaction with *β*‐TrCP, we generated two truncated mutants of OTUD6B: N‐terminal OTUD6B, which contains the coiled‐coil domain, and C‐terminal OTUD6B, which includes the amino‐terminal OTU domain. Co‐immunoprecipitation assay showed that N‐terminal OTUD6B mediated the interaction with *β*‐TrCP (Figure 2F). In addition, we also generated two truncated mutants of *β*‐TrCP: N‐terminal *β*‐TrCP, which contains the F‐box domain, and C‐terminal *β*‐TrCP, which includes 7 WD‐40 domains. Since the N‐terminal deletion mutant of *β*‐TrCP was unable to interact with OTUD6B (Figure 2G), we reasoned that OTUD6B N‐terminal domain and *β*‐TrCP N‐terminal domain mediated their direct interaction.

We then performed ubiquitination assays to examine whether OTUD6B stabilizes *β*‐TrCP protein through deubiquitination. First, an in vitro ubiquitination assay demonstrated that OTUD6B was capable of cleaving Ub(n)‐ubiquitinylated substrates similar to other DUBs such as USP2, though it takes a longer time (Figure 2H and Figure S2B,C, Supporting Information). More importantly, OTUD6B markedly reduced *β*‐TrCP ubiquitination in vitro (Figure 2I), indicating it functions as a DUB for *β*‐TrCP.

Second, OTUD6B significantly reduced the ubiquitination of *β*‐TrCP in vivo, while OTUD6B^C188S^ did not show the same effect (Figure 2J,K and Figure S2E, Supporting Information). Again, the effect of OTUD6B was much stronger than that of USP47 in reducing the ubiquitination of *β*‐TrCP (Figure S2D, Supporting Information), suggesting OTUD6B functions as a major DUB for *β*‐TrCP. Moreover, the ubiquitylation of *β*‐TrCP notably increased in OTUD6B‐knockdown cells compared to control cells (Figure 2L and Figure S2F, Supporting Information). The ubiquitination of *β*‐TrCP is specifically regulated by OTUD6B but no other OTU family members, since the ubiquitylation status of *β*‐TrCP did not change in OTUD6A‐ or OTUD2‐knockdown cells (Figure S2G, Supporting Information). In addition, OTUD6B specifically removed the K48 ubiquitin chain (Figure 2M), which mediate protein stability,^[^ ^13^ ^]^ further supporting the notion that OTUD6B stabilizes *β*‐TrCP protein via deubiquitination. Collectively, these results suggest that OTUD6B functions as a bona fide DUB that directly targeting and stabilizing *β*‐TrCP.

### 2.3 OTUD6B Reduces Tumor‐Initiating Cell Properties of Esophageal Squamous Cell Carcinoma Cells via *β*‐TrCP

**Figure 3:**
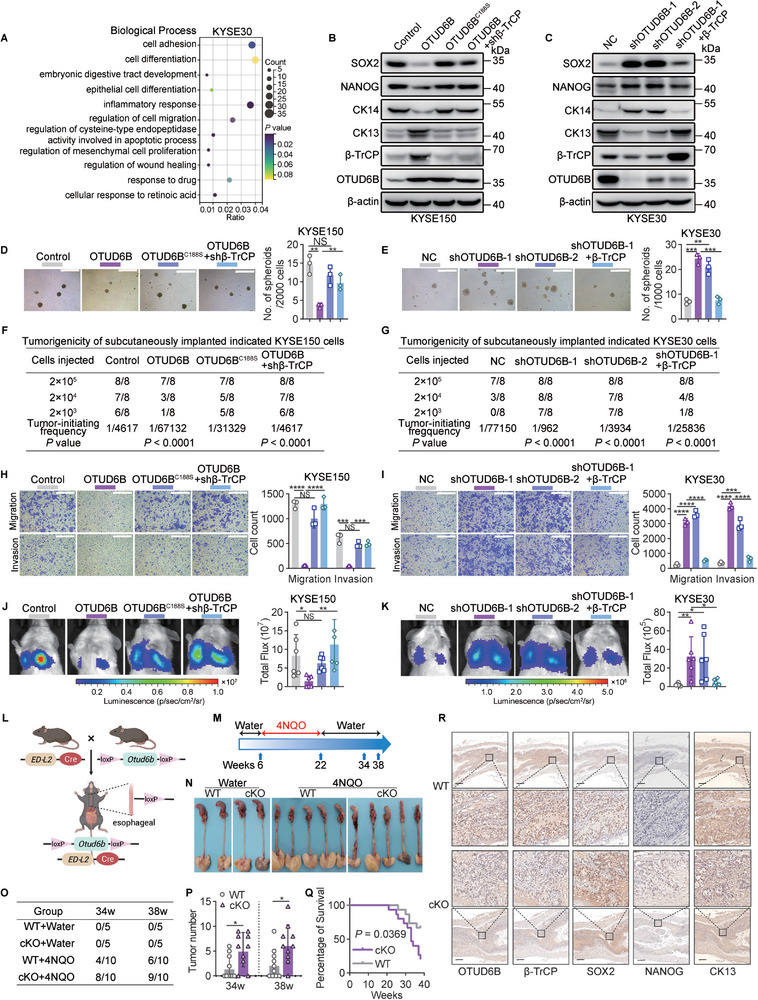
OTUD6B reduces TIC properties of ESCC cells via *β*‐TrCP. A) KYSE30 cells with or without OTUD6B‐knockdown were subjected to RNA‐seq and gene ontology (GO) analysis. A bubble chart shows the partial enriched terms of biological processes. The RNA‐sequencing data are available at the Gene Expression Omnibus (GEO) database (accession GSE209521). B) The levels of SOX2, NANOG, CK14, and CK13 in the indicated KYSE150 cells and C) KYSE30 cells. Data from a representative experiment in at least two replicates. D) The spheroid formation capacity of the indicated KYSE150 cells and E) KYSE30 cells. Scale bars, 500 µm. *n* = 3, mean ± SEM, unpaired *t*‐test, ** *p* < 0.01, *** *p* < 0.001, NS, not significant (*p* > 0.05). F) The indicated KYSE150 cells and G) KYSE30 cells were subcutaneously injected into BALB/c nude mice to detect the tumor formation rates. H) The migration and invasion capabilities of the indicated KYSE150 cells and I) KYSE30 cells. Scale bars, 500 µm. *n* = 3, mean ± SEM, unpaired *t*‐test, *** *p* < 0.001, **** *p* < 0.0001, NS, not significant (*p* > 0.05). J) The indicated KYSE150 cells and K) KYSE30 cells were injected into the tail vein of NOD/SCID mice. Lung metastasis was detected by bioluminescence imaging. *n* = 6, mean ± SD, unpaired *t*‐test, * *p* < 0.05, ** *p* < 0.01, NS, not significant (*p* > 0.05). L) A model of *Otud6b* esophageal epithelium‐specific conditional knockout (cKO) mice. M) A schematic diagram shows the 4NQO‐induced carcinogenesis model. N) Representative images of the esophagus in 4NQO‐induced *Otud6b* WT and cKO mice. O) The tumor formation rates and P) tumor number of the esophagus in 4NQO‐induced *Otud6b* WT and cKO mice. *n* = 10, mean ± SD, unpaired *t*‐test, * *p* < 0.05. Q) The overall survival of *Otud6b* WT and cKO mice treated with 4NQO. *n* = 15, Pearson's test. R) IHC staining of the indicated proteins in the esophagus of 4NQO‐induced *Otud6b* WT and cKO mice.

Given the important roles of *β*‐TrCP in ESCC development, we wonder if OTUD6B also plays a major role in regulating ESCC progression. To explore the functions of OTUD6B in ESCC cells, we performed RNA sequencing on ESCC cells with or without OTUD6B‐knockdown. Gene ontology (GO) analysis revealed that cell differentiation and cell migration were significantly influenced by OTUD6B‐knockdown (**Figure** **3**A and Figure S3A, Supporting Information). These results suggest that OTUD6B may regulate stemness and TIC properties of ESCC cells. Consistent with this note, we found that the expression levels of several key stem cell markers, including SOX2, NANOG, and CK14, were significantly decreased upon OTUD6B overexpression but increased when OTUD6B was knocked down; while the esophageal epithelial differentiation marker CK13 was positively correlated with OTUD6B expression (Figure 3B,C and Figure S3B,C, Supporting Information). OTUD6B regulates these stemness markers via a *β*‐TrCP‐dependent manner, since this effect was rescued by the knockdown of *β*‐TrCP (Figure 3B,C and Figure S3B,C, Supporting Information).

In addition, spheroid formation capacity was inhibited in KYSE150 and KYSE180 cells by WT OTUD6B overexpression but not OTUD6B^C188S^, while the capacity was increased in OTUD6B‐knockdown KYSE30 and KYSE450 cells (Figure 3D,E and Figure S3D,E, Supporting Information). Limiting dilution assay revealed that overexpression of OTUD6B but not OTUD6B^C188S^ decreased the frequency of ESCC TICs in vivo, whereas knockdown of OTUD6B increased the frequency (Figure 3F,G and Figure S3F,G, Supporting Information). The ESCC metastasis was also inhibited by overexpressing OTUD6B but not OTUD6B^C188S^, but increased by knockdown of OTUD6B (Figure 3H–K and Figure S3H–K, Supporting Information). In addition, all the effects of OTUD6B overexpression or knockdown in ESCC cells were rescued by the knockdown or overexpression of *β*‐TrCP respectively (Figure 3D–K and Figure S3D–K, Supporting Information). Taken together, all these results indicate an important role of OTUD6B in suppressing ESCC stemness and progression in a *β*‐TrCP‐dependent manner.

To further investigate the role of OTUD6B in regulating ESCC progression in an immune‐competent setting, we generated a conditional knockout (cKO) mouse model in which *Otud6b* gene can be specifically targeted in the esophageal epithelial tissue (Figure 3L and Figure S3L, Supporting Information). There were no significant differences in body and organ size between *Otud6b^LoxP/LoxP^*; *ED‐L2‐Cre^+^* (*Otud6b* cKO) mice and *Otud6b^LoxP/LoxP^; ED‐L2‐Cre^−^* (*Otud6b* WT) mice (Figure S3M,N, Supporting Information). The expression of OTUD6B was knocked out in the esophagus of *Otud6b* cKO mice but not in other organs (Figure S3O, Supporting Information). To explore the role of OTUD6B in regulating ESCC progression, we induced ESCC tumors by 4‐nitroquinoline 1‐oxide (4NQO) in *Otud6b* WT and cKO backgrounds^[^ ^14^ ^]^ (Figure 3M). Knockout of OTUD6B significantly promoted the development of esophageal carcinoma in the 4NQO‐induced model (Figure 3N and Figure S3P, Supporting Information). Compared to *Otud6b* WT mice, *Otud6b* cKO mice had a higher incidence of induced tumors in the esophageal epithelium (Figure 3O,P). In the 4NQO‐treated groups, the body weight of *Otud6b* cKO mice was significantly lower than that of wild‐type mice (Figure S3Q, Supporting Information). The survival of *Otud6b* cKO mice was significantly poorer than that of *Otud6b* WT mice (Figure 3Q). We did not find spontaneous tumorigenesis in *Otud6b* cKO mice until 38 weeks, indicating that loss of OTUD6B alone is not sufficient to induce tumorigenesis in esophageal epithelial tissue. Moreover, in the induced esophageal tumors of *Otud6b* cKO mice, the expression of *β*‐TrCP and the differentiation marker CK13 was decreased, and the levels of SOX2 and NANOG were increased (Figure 3R). These results indicate that in an immune‐competent setting, OTUD6B still functions as an important tumor suppressor of ESCC development.

### 2.4 OTUD6B Promotes the Degradation of SNAIL

**Figure 4:**
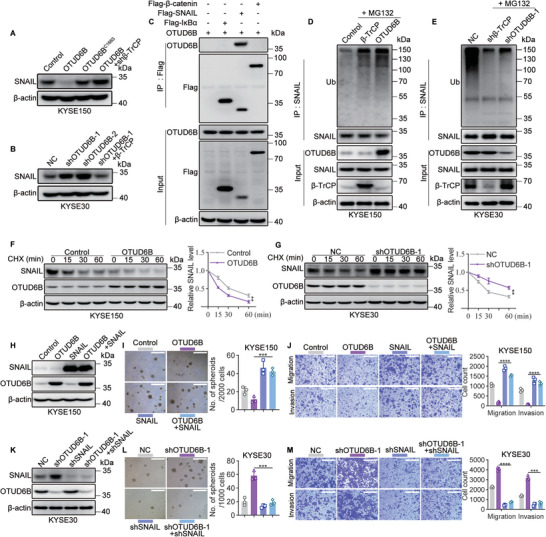
OTUD6B promotes the degradation of SNAIL. A) The levels of SNAIL in the indicated KYSE150 cells and B) KYSE30 cells were detected by immunoblotting. C) Flag‐*β*‐catenin, flag‐SNAIL, or flag‐I*κ*B*α* was co‐transfected with OTUD6B into HEK293T cells. Their interactions were detected by immunoprecipitation assay. D) The ubiquitination level of SNAIL in OTUD6B‐ or *β*‐TrCP‐ overexpressing KYSE150 cells. E) The ubiquitination level of SNAIL in *β*‐TrCP‐knockdown or OTUD6B‐knockdown KYSE30 cells. F) The degradation of SNAIL in OTUD6B‐overexpressing KYSE150 cells treated with CHX (50 µg mL^−1^) for 15, 30, 60 min. *n* = 3, mean ± SD, unpaired *t*‐test, ** *p* < 0.01. G) The degradation of SNAIL in OTUD6B‐knockdown KYSE30 cells treated with CHX (50 µg mL^−1^) for 15, 30, 60 min. *n* = 3, mean ± SD, unpaired *t*‐test, ** *p* < 0.01. H) SNAIL was overexpressed in OTUD6B‐overexpressing or control KYSE150 cells. The levels of SNAIL and OTUD6B were detected by immunoblotting. I) The spheroid formation capacity and J) migration and invasion capabilities of the indicated KYSE150 cells. Scale bars, 500 µm. *n* = 3, mean ± SEM, unpaired *t*‐test, *** *p* < 0.001, **** *p* < 0.0001. K) SNAIL was knocked down in OTUD6B‐knockdown KYSE30 cells. The levels of SNAIL and OTUD6B were detected by immunoblotting. L) The spheroid formation capacity and M) migration and invasion capabilities of the indicated KYSE30 cells were detected. Scale bars, 500 µm. *n* = 3, mean ± SEM, unpaired *t*‐test, *** *p* < 0.001, **** *p* < 0.0001. The data shown in (A)–(G) are from a representative experiment in at least two replicates.

As an E3 ligase, *β*‐TrCP mediates the degradation of several substrate proteins through the UPS, including the EMT‐TF SNAIL that plays an importance role in regulating cancer stemness.^[^ ^6^, ^15^ ^]^ In addition, the expression of SNAIL was significantly inhibited upon OTUD6B overexpression but increased when OTUD6B was knocked down, and these effects were mediated in a *β*‐TrCP‐dependent manner (**Figure** **4**A,B and Figure S4A,B, Supporting Information). Next, we performed a coimmunoprecipitation assay to test the interaction between OTUD6B and several known *β*‐TrCP substrate proteins, including SNAIL, *β*‐catenin and I*κ*B*α*. The results showed that OTUD6B could bind with SNAIL but not *β*‐catenin or I*κ*B*α* (Figure 4C). Thus, we speculated that suppression the TIC properties of ESCC cells by OTUD6B might be caused by decreased SNAIL stability in a *β*‐TrCP‐dependent manner. As expected, overexpression of OTUD6B increased, while knockdown of OTUD6B decreased, the ubiquitination level and degradation of SNAIL in ESCC cells (Figure 4D–G and Figure S4C–F, Supporting Information). Moreover, overexpression of SNAIL reversed the inhibition of spheroid formation capacity and cell migration and invasion ability in OTUD6B‐overexpressing cells; knockdown of SNAIL reversed the increase in the TIC properties of OTUD6B‐knockdown cells (Figure 4H–M and Figure S4G–L, Supporting Information). Thus, these results demonstrated that OTUD6B inhibited TIC properties of ESCC cells by decreasing SNAIL stability.

### 2.5 Reduced OTUD6B Predicts a Poor Prognosis in Esophageal Squamous Cell Carcinoma Patients

**Figure 5:**
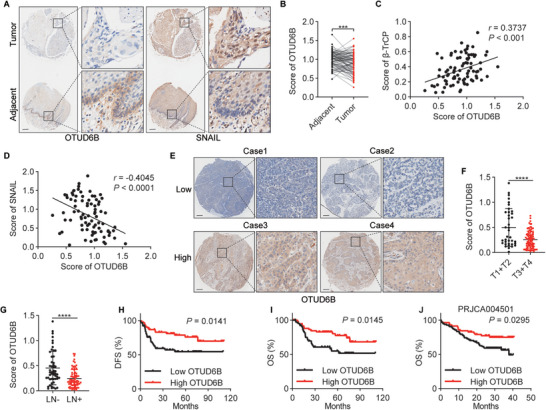
Reduced OTUD6B predicts a poor prognosis in ESCC patients. A) The expression of OTUD6B and SNAIL in 85 paired ESCC and adjacent normal tissues was detected by immunohistochemistry, and B) the relative expression of OTUD6B was analyzed. Paired *t*‐test, *** *p* < 0.001. C) The expression of OTUD6B is positively correlated with *β*‐TrCP and D) negative correlated with SNAIL in ESCC samples. Pearson's test. E) The expression of OTUD6B in 128 ESCC tissues was detected by immunohistochemistry, and F) stratified by tumor pathological stage or G) lymph node metastasis. unpaired *t*‐test, **** *p* < 0.0001. H) Kaplan‐Meier plot of disease‐free survival and I) overall survival of 128 ESCC patients stratified by OTUD6B expression. Two‐sided log‐rank test. J) Kaplan‐Meier plot of the overall survival of ESCC patients stratified by OTUD6B expression using the PRJCA004501 datasets. Two‐sided log‐rank test.

We next examined the clinical importance of the OTUD6B‐*β*‐TrCP‐SNAIL axis and their predictive power of ESCC prognosis. OTUD6B was decreased in ESCC tissues compared to their adjacent normal tissues (**Figure** **5**A,B). In support of the notion that OTUD6B enhances *β*‐TrCP protein stability and thus decreases SNAIL protein level, we found a significant positive correlation between OTUD6B and *β*‐TrCP protein levels in ESCC tissues (Figure 5C) and a negative correlation between OTUD6B and SNAIL protein levels (Figure 5D). Additionally, we thoroughly analyzed the relationship between OTUD6B protein levels and several key pathological parameters in 128 ESCC specimens (Figure 5E). The statistical analyses showed that patients with advanced pathological stages displayed a lower OTUD6B expression (Figure 5F). A lower OTUD6B level was observed in patients with lymph node metastasis compared with those without metastasis (Figure 5G). Importantly, low OTUD6B protein level significantly correlates with poor disease‐free survival and overall survival (OS) (Figure 5H,I). This correlation is further confirmed using other public datasets of ESCC samples (Figure 5J and Figure S5A,B, Supporting Information).

### 2.6 OTUD6B is Critical for All‐Trans Retinoic Acid‐Mediated Inhibition of Tumor‐Initiating Cell Properties in Esophageal Squamous Cell Carcinoma

**Figure 6:**
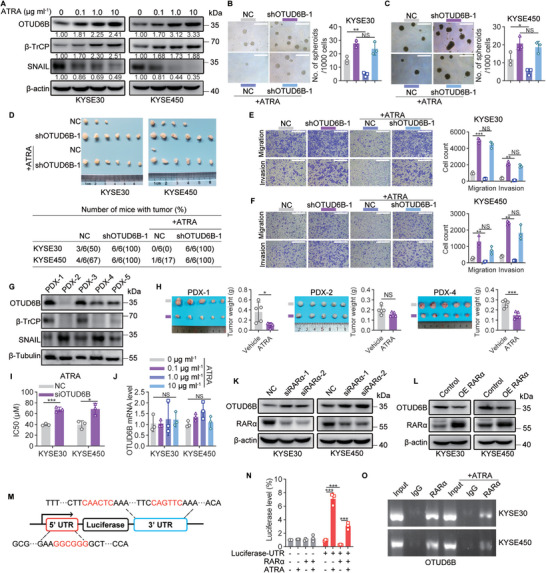
OTUD6B is critical for ATRA‐mediated inhibition of TIC properties in ESCC. A) The protein levels of OTUD6B, *β*‐TrCP, and SNAIL in KYSE30 and KYSE450 cells treated with ATRA (0.1, 1, 10 µg mL^−1^). B) The spheroid formation capacity of OTUD6B‐knockdown KYSE30 and C) KYSE450 cells treated with or without ATRA (1 µg mL^−1^). Scale bars, 500 µm. *n* = 3, mean ± SEM, unpaired *t*‐test, * *p* < 0.05, ** *p* < 0.01, NS, not significant (*p* > 0.05). D) The indicated KYSE30 or KYSE450 cells (2 × 10^4^) were subcutaneously injected into BALB/c nude mice treated with or without ATRA (5 mg kg^−1^) to detect the tumor formation rates. E) The migration and invasion capabilities of the indicated KYSE30 cells or F) KYSE450 cells treated with or without ATRA (1 µg mL^−1^). Scale bars, 500 µm. *n* = 3, mean ± SEM, unpaired *t*‐test, ** *p* < 0.01, *** *p* < 0.001, NS, not significant (*p* > 0.05). G) The expression level of OTUD6B, *β*‐TrCP, and SNAIL in tumor tissues of five ESCC patient‐derived xenograft (PDX) samples. H) Tumor images and tumor weight of the PDX‐1, PDX‐2, and PDX‐4 groups treated with or without ATRA (5 mg kg^−1^). *n* = 3, mean ± SD, unpaired *t*‐test, * *p* < 0.05, *** *p* < 0.001, NS, not significant (*p* > 0.05). I) The half maximal inhibitory concentration (IC50) of ATRA in OTUD6B‐knockdown and control KYSE30 or KYSE450 cells were analyzed in the genomics of drug sensitivity in cancer (GDSC) database using RNA‐sequencing data. *n* = 3, mean ± SD, unpaired *t*‐test, * *p* < 0.05, *** *p* < 0.001. J) The mRNA level of OTUD6B in KYSE30 and KYSE450 cells treated with ATRA (0.1, 1, 10 µg mL^−1^). *n* = 3, mean ± SEM, one‐way ANOVA test, NS, not significant (*p* > 0.05). K) The protein level of OTUD6B was detected in RAR*α*‐knockdown or L) RAR*α*‐overexpressing KYSE30 and KYSE450 cells. M) A schematic diagram shows the luciferase reporter plasmid containing the 5’ UTR and 3’ UTR of OTUD6B mRNA. The target sequences of RAR*α* are shown in red. N) HEK293T cells were transfected with the indicated plasmids, and treated with or without ATRA (1 µg mL^−1^). The firefly luciferase activity was detected and normalized to the renilla luciferase activity. *n* = 3, mean ± SEM, unpaired *t*‐test, *** *p* < 0.001. O) KYSE30 and KYSE450 cells were treated with or without ATRA (1 µg mL^−1^) followed by RIP assay using anti‐ RAR*α* or IgG antibodies. The data shown in (A), (G), (K), and (L) are from a representative experiment in at least two replicates.

When comparing the transcriptomes between OTUD6B‐knockdown and control ESCC cells, a number of genes associated with the cellular response to retinoic acid were found to show significant differences (Figure 3A and Figure S3A, Supporting Information). ATRA is known to induce differentiation and inhibit stemness in multiple cell types,^[^ ^16^ ^]^ and involved in the downregulation of some *β*‐TrCP substrate proteins, including TWIST, MYC, CCND1, and CTNNB1.^[^ ^17^ ^]^ Thus, we speculated that ATRA may selectively target ESCC cancer cells and suppress their TIC properties. To verify this hypothesis, we treated KYSE30 and KYSE450 cells with ATRA and examined the expression change of OTUD6B protein. Indeed, ATRA treatment significantly increased OTUD6B protein level in a dose‐dependent manner and showed little effect on OTUD6A or OTUD2 expression (**Figure** **6**A and Figure S6A, Supporting Information). Consistent with the notion that OTUD6B enhances *β*‐TrCP protein stability and decreases SNAIL protein level, ATRA treatment upregulated the level of *β*‐TrCP and downregulated the expression of SNAIL (Figure 6A). Moreover, ATRA treatment could significantly decrease spheroid formation capacity, tumorigenic ability, and cell migration and invasion ability of ESCC cells (Figure 6B–F). In ESCC patient‐derived xenograft (PDX) models, the inhibitory effect of ATRA on tumor growth was also observed, and the level of OTUD6B was positively correlated with *β*‐TrCP and negatively correlated with SNAIL (Figure 6G,H and Figure S6B, Supporting Information). Taken together, these results showed that ATRA upregulated the protein level of OTUD6B in ESCC cells and inhibited their TIC properties.

Interestingly, we noted that the effects of ATRA in suppressing TIC properties were diminished in OTUD6B‐knockdown cells (Figure 6B–F). Consistently, a drug sensitivity prediction analysis using the genomics of drug sensitivity in cancer database found that the sensitivity of OTUD6B‐knockdown cells to ATRA was likely to be significantly lower than that of control cells (Figure 6I). Moreover, among the PDX models examined, only the OTUD6B‐expression ones showed an obvious response to ATRA treatment (Figure 6G,H). To better understand these observations, we further explored the mechanism how ATRA regulates OTUD6B expression. First, we found that mRNA level of OTUD6B in ESCC cells treated with ATRA did not change (Figure 6J), suggesting that ATRA may upregulate OTUD6B protein via regulating its translation. Thus, in cancer cells lacking OTUD6B transcripts, ATRA would show little effects on promoting OTUD6B protein level and suppressing their TIC properties. Since RAR*α* has been reported to repress the translation of specific mRNAs through binding with the untranslated region (UTR) of target mRNA, and this repression can be relieved by ATRA,^[^ ^18^ ^]^ we tested whether ATRA promoted the translation of OTUD6B by relieving the binding of RAR*α* to the UTR of OTUD6B mRNA. We found that knockdown of RAR*α* significantly increased the protein level of OTUD6B in ESCC cells, while ectopic expression of RAR*α* decreased it (Figure 6K,L). In addition, both the 5’ UTR and 3’ UTR of OTUD6B mRNA contained the specific motif for RAR*α* binding (Figure 6M). Using a luciferase reporter containing 5’ UTR and 3’ UTR of OTUD6B (Figure 6M), we found that ATRA promotes the luciferase translation in a RAR*α*‐dependent manner, confirming it recognizes OTUD6B mRNA and thus regulates its translation (Figure 6N). Importantly, RNA‐immunoprecipitation (RIP) revealed that RAR*α* bound with OTUD6B mRNA, and ATRA treatment reduced this binding activity (Figure 6O). Collectively, these results indicated that ATRA promoted the translation of OTUD6B via relieving RAR*α* binding to its mRNAs and thus repressed the TIC properties of ESCC cells.

### 2.7 ATRA Enhances the Response of Established Esophageal Squamous Cell Carcinoma Tumors to Anti‐PD‐1 Immunotherapy

**Figure 7:**
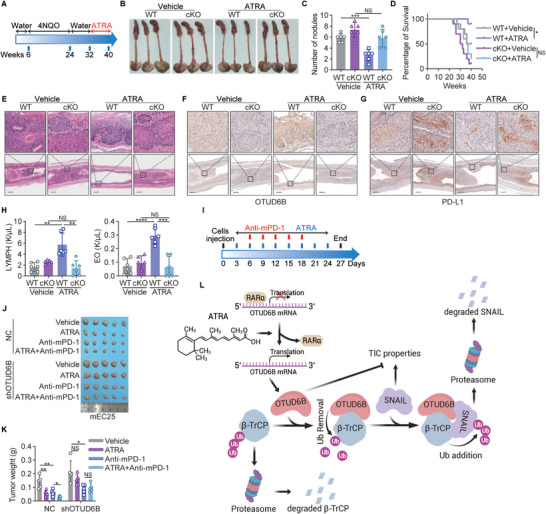
ATRA enhances the response of established ESCC tumors to anti‐PD‐1 immunotherapy. A) A schematic diagram shows 4NQO‐induced carcinogenesis in the ATRA treated model. B) The representative images and C) tumor number of the esophagus in 4NQO‐induced *Otud6b* WT and cKO mice treated with or without ATRA. *n* = 6, mean ± SD, unpaired *t*‐test, *** *p* < 0.001, NS, not significant (*p* > 0.05). D) The overall survival of 4NQO‐induced *Otud6b* WT and cKO mice treated with or without ATRA. *n* = 10, Pearson's test, * *p* < 0.05, NS, not significant (*p* > 0.05). E) H&E and F) IHC staining of OTUD6B and G) PD‐L1 in the esophagus of 4NQO‐induced *Otud6b* WT and cKO mice treated with or without ATRA. H) The numbers of LYMPHs and EOs in the peripheral blood of 4NQO‐induced *Otud6b* WT and cKO mice treated with or without ATRA. *n* = 6, mean ± SD, unpaired *t*‐test, ** *p* < 0.01, *** *p* < 0.001, **** *p* < 0.0001, NS, not significant (*p* > 0.05). I) A schematic diagram shows the ATRA and mouse anti‐PD‐1 (anti‐mPD‐1) combination therapy. J) The tumor images and K) tumor weigh of the indicated mEC25 cells with or without knockdown of OTUD6B. *n* = 5, mean ± SD, unpaired *t*‐test, * *p* < 0.05, ** *p* < 0.01, NS, not significant (*p* > 0.05). L) A model shows that ATRA promotes the translation of OTUD6B which plays an important role in suppression of the TIC properties of ESCC through regulating ubiquitination of *β*‐TrCP and SNAIL. The figure was created with BioRender.com.

To further investigate the effects of ATRA on treating ESCC tumors in an immune‐competent setting, we treated 4NQO‐induced *Otud6b* cKO and WT mice with ATRA (**Figure** **7**A). There were no significant differences in body size and organs of mice treated with or without ATRA, suggesting limited side‐effects of this treatment (Figure S7A,B, Supporting Information). ATRA treatment significantly reduced the number of induced tumors in *Otud6b* WT mice but not in *Otud6b* cKO mice (Figure 7B,C), supporting the notion that ATRA relies on OTUD6B mRNA to suppress ESCC progression. In addition, the survival of ATRA‐treated *Otud6b* WT tumor‐bearing mice was significantly higher than that of untreated mice, but there was no significant difference in *Otud6b* cKO mice (Figure 7D). Compared to untreated group, the expression level of OTUD6B in ESCC tumors was significantly increased in ATRA‐treated *Otud6b* WT mice (Figure 7E,F).

It has been reported that ATRA can enhance the efficacy of the anti‐PD‐1 antibody pembrolizumab in stage IV melanoma patients,^[^ ^19^ ^]^ and PD‐L1 has been reported as a substrate of *β*‐TrCP.^[^ ^20^ ^]^ In addition, in *Otud6b* WT mice, we found that ATRA treatment significantly reduced the expression levels of PD‐L1, but not in *Otud6b* cKO mice (Figure 7G). Moreover, in ATRA‐treated *Otud6b* WT mice, we noticed an increase of lymphocytes (LYMPHs) and eosinophils (EOs) counts in the peripheral blood (Figure 7H), which has been reported to associate with favorable OS of patients treated with anti‐PD‐1 antibodies.^[^ ^21^ ^]^ Thus, we treated mice bearing xenograft tumors initiated by mEC25, a mouse ESCC cell line, with ATRA and anti‐PD‐1 antibody to detect the role of ATRA in the anti‐PD‐1 immunotherapy. As shown in Figure 7I–K, combination of ATRA and anti‐PD‐1 antibody displayed more potent effects in suppressing tumor growth than either of the single agent. The ATRA‐mediated enhancement of tumor response to anti‐PD‐1 treatment relies on the existence of OTUD6B mRNA, since in OTUD6B‐knockdown ESCC tumors, ATRA could not improve the effects of anti‐PD‐1 immunotherapy (Figure 7J,K). Taken together, these results demonstrate the potential to develop combination therapy using ATRA and anti‐PD‐1 antibodies for a cohort of OTUD6B‐expression ESCC patients.

## 3 Discussion

*β*‐TrCP is a key E3 ubiquitin ligase and plays important roles in multiple human cancers including ESCC, and increases the ubiquitination level and stability of SNAIL which plays an importance role in regulating cancer stemness.^[^ ^5^, ^6^, ^15^ ^]^ In this study, we identified OTUD6B as a potent DUB of *β*‐TrCP that promotes *β*‐TrCP protein stability via suppressing its ubiquitination and degradation, and interacts with SNAIL to increase the ubiquitination level and stability of SNAIL by *β*‐TrCP. Knockdown of OTUD6B increases SNAIL expression through decreasing *β*‐TrCP protein stability, and accelerates ESCC tumor progression through the OTUD6B‐*β*‐TrCP‐SNAIL axis (Figure 7L). Moreover, low OTUD6B expression is significantly correlated with decreased *β*‐TrCP and increased SNAIL protein level in ESCC tissues and predicts a poor prognosis in ESCC patients. Importantly, we found that ATRA treatment could efficiently suppress ESCC tumor initiation and progression through promoting the translation of OTUD6B. In immune‐competent mouse models, ATRA treatment enhances the response of established ESCC tumors to anti‐PD‐1 immunotherapy, implying that combination therapies using ATRA and immune checkpoint inhibitors may be a better therapeutic option for certain ESCC patients.

OTUD6B is a member of the ovarian tumor protease (OTU) family and was initially considered as an inactive DUB.^[^ ^22^ ^]^ It has been reported to regulate protein synthesis in non‐small cell lung cancer cells^[^ ^23^ ^]^ and inhibit hepatocellular carcinoma metastasis independent of its catalytic activity.^[^ ^24^ ^]^ Here we show that OTUD6B reduces Ub(n)‐ubiquitinylated substrates, especially polyubiquitin chains, demonstrating that it is a bona fide DUB. The catalytic activity of OTUD6B is essential to regulate *β*‐TrCP and SNAIL protein stability and to suppress the TIC properties of ESCC cells.

ATRA has been reported for its function in treating acute promyelocytic leukaemia patients.^[^ ^25^ ^]^ An increasing number of studies have demonstrated that ATRA inhibits TIC properties in various solid cancer types.^[^ ^26^ ^]^ For example, ATRA promotes the differentiation of GBM stem cells and thus suppresses tumor growth by regulating the Notch pathway.^[^ ^26b^ ^]^ Here, we found that ATRA suppressed ESCC tumor initiation and progression by promoting OTUD6B translation and inhibiting TIC properties of ESCC cancer cells. Importantly, ATRA could increase the response of established ESCC tumors to anti‐PD‐1 immunotherapy. Given the fact that only a proportion of ESCC patients respond to immune checkpoint inhibitors, combination of ATRA and anti‐PD‐1 hold the promise to increase the percentage of ESCC patients to benefit from anti‐PD‐1 immunotherapy. Moreover, we found that ATRA promotes OTUD6B translation by relieving RAR*α* binding to its mRNAs. As a result, the tumor suppressive effects of ATRA require the existence of OTUD6B transcripts, which suggests that OTUD6B transcript level can serve as a biomarker to predict whether ATRA would benefit specific ESCC patients.

## 4 Experimental Section

### Cell Culture

The human ESCC cell lines KYSE series were generously provided by Dr. Shimada Y. and cultured in RPMI/1640 medium (HyClone, USA) supplemented with 10% fetal bovine serum (HyClone, USA).^[^ ^27^ ^]^ The HEK293T cell line was purchased from American Type Culture Collection (ATCC) and cultured in DMEM (HyClone, USA) plus 10% fetal bovine serum. The mEC25 cell line was generously provided by Dr. Li Fu and cultured in DMEM (HyClone, USA) plus 10% fetal bovine serum.^[^ ^28^ ^]^ All cell lines were validated by short tandem repeat (STR) DNA fingerprinting and confirmed to free of mycoplasma contamination.

### Plasmids, Reagents, and Antibodies

OTUD6B, OTUD6B^C188S^, *β*‐TrCP and SNAIL were inserted into the pLVX‐IRES vector. The OTUD6B and *β*‐TrCP truncated mutants were cloned into the pEBG‐GST vector. RAR*α* was inserted into pENTER vector. The DUBs (including ATXN3, BRCC3, COPS5, USP15, USP47, UCHL1, OTUB1, OTUD6B, and VCPIP1) were cloned into the pcDNA3 vector. The OTUD6B shRNA, *β*‐TrCP, and SNAIL shRNA sequences were cloned into the pSIH‐H1 vector. The siRNAs targeting RAR*α* were purchased from RiboBio (Guangzhou, China). All primers used for plasmid construction and the target sequences of shRNAs and siRNAs are shown in Table S1, Supporting Information.

The reagents were as follows: ATRA (Sigma, USA), MG132 (Sigma, USA), CHX (Sigma, USA), cisplatin (Sigma, USA), 4‐nitroquinoline 1‐oxide (4NQO, Sigma, USA), XenoLight D‐Luciferin K+ salt (PerkinElmer, USA), TRIzol (Invitrogen, USA), puromycin (Sangon Biotech, China), G418 (Sigma, USA), 4,6‐diamidino‐2‐phenylindole (DAPI, Sangon Biotech, China).

The information on antibodies used in this study is provided in Table S2, Supporting Information.

### GST‐Tagged Protein Purification

OTUD6B was cloned into the bacterial expression vector pGEX‐6P‐1. GST‐OTUD6B was expressed in *E. coli* BL21 (DE3) under 0.5 mm IPTG at 37 °C. Cells were harvested and resuspended in lysis buffer (50 mm Tris‐HCl, 150 nM NaCl, pH 8.0) followed by sonication. The target proteins were obtained by one‐step purification using a GST column (L00206, GeneScript).

### GST Pull‐Down Assay

Cells were harvested and resuspended in cell lysis buffer (10 mm Tris‐HCl pH 7.5, 150 mm NaCl, 5 mm EDTA, 1% Triton X‐100). The cell lysates were incubated with GST‐OTUD6B or GST protein at 4 °C overnight and then incubated with GST agarose for 4 h. The beads were washed with lysis buffer three times, and the immunoprecipitates were analyzed by immunoblotting. The GST proteins were stained with Coomassie Blue.

### Cycloheximide Pulse‐Chase Assay

For the protein half‐life assay, the indicated cells were treated with CHX (50 µg mL^−1^) for the indicated time points, and subjected to immunoblotting.

### Immunoprecipitation and In Vivo Ubiquitination Assay

For immunoprecipitation and in vivo ubiquitination assays, the indicated cells were treated with MG132 (20 µM) for 6 h and lysed with cell lysis buffer. The lysates were incubated with anti‐Flag M2 affinity gel or indicated antibodies with protein A/G magnetic beads overnight at 4 °C. After washing with lysis buffer, the immunoprecipitates were used for immunoblotting with the indicated antibodies.

### In Vitro Ubiquitination Assay

For the in vitro ubiquitination assay, the Ub(n)‐ubiquitinylated substrate (ab157043, Abcam) was incubated with recombinant GST protein, recombinant GST‐OTUD6B protein or USP2 protein in deubiquitination reaction buffer (50 mm HEPES, pH 7.5, 100 mm NaCl, 5 mm MgCl_2_, 1 mm ATP, 1 mm DTT, 5% glycerol) for the indicated durations at 37 °C. The proteins were subjected to immunoblotting for anti‐Ubiquitin and stained with Coomassie Blue. For *β*‐TrCP in vitro ubiquitination assay, HEK293T cells were plated into six‐well plates and co‐transfected with Flag‐*β*‐TrCP and HA‐ubiquitin. After cells were treated with MG132 (20 µM) for 6 h, ubiquitylated *β*‐TrCP was purified by anti‐Flag M2 affinity gel. The purified ubiquitylated *β*‐TrCP was incubated with recombinant GST protein or recombinant GST‐OTUD6B protein in deubiquitination reaction buffer for 2 h at 37 °C. The beads were washed with PBS three times, and the ubiquitinated status of *β*‐TrCP was analyzed by immunoblotting.

### Immunofluorescence Staining

The indicated cells were seeded into a µ‐Slide VI chamber (ibidi, Germany). After fixation with 4% paraformaldehyde and permeabilization with Triton X‐100, adhered cells were blocked with 5% BSA and incubated with primary antibodies. The protein signals were visualized by anti‐rabbit IgG conjugated with Alexa Fluor 488 (#4416, Cell Signaling Technology, USA), anti‐mouse IgG conjugated with Alexa Fluor 555 (#4409, Cell Signaling Technology, USA), and the nuclear dye 4,6‐diamidino‐2‐phenylindole (DAPI). The cells were imaged using a laser confocal microscope.

### Spheroid Formation Assay

The indicated cells were plated into 24‐well low attachment plates (#3474, Corning, USA) and cultured in serum‐free DMEM/F‐12 medium (Gibco, USA) supplemented with B27 (1:50, Gibco), bFGF (10 ng mL^−1^, Invitrogen), hEGF (20 ng mL^−1^, Gibco), and insulin (4 µg mL^−1^, Gibco). Fresh medium was added every 2 days. After 2 weeks, spheres with a diameter of at least 100 µm were counted manually.

### Migration and Invasion Assays

The indicated cells suspended in serum‐free medium were plated onto Boyden chambers (3422, Corning Incorporated, USA) with or without coated Matrigel (Corning Incorporated, USA). The bottom chambers were filled with 10% fetal bovine serum medium. After 24 h, the bottom cells were fixed and stained with crystal violet, and cells in three randomly selected views were photographed and counted.

### Immunohistochemical Staining

85 paired human ESCC and adjacent normal tissues were obtained from Nanjing First Hospital, and 128 other ESCC specimens were collected from Zhejiang Cancer Hospital. No patients received chemotherapy or radiotherapy before surgery or had postoperative complications after surgery. The experiments were approved by the ethical committee of the hospitals, and all patients provided informed consent. Immunohistochemistry staining was performed following the immunoperoxidase method. First, the sections were dewaxed in xylene and dehydrated in alcohol. After antigen retrieval, the tissues were blocked with goat serum albumin at room temperature for 1 h and incubated with primary antibody at 4 °C overnight. Finally, the slides were incubated with a secondary antibody, stained with diaminobenzidine, and counterstained with haematoxylin. All slides were scanned by an Aperio ImageScope system (Leica Biosystems, USA) and scored according to the intensity of the stained cells. The values were as follows: Negative staining, 0; weak positive, 1; positive, 2; and strong positive, 3.

### Quantitative Real‐Time PCR

Total RNA was extracted from the indicated cells using TRIzol reagent (Thermo Scientific, USA) and reverse transcribed using the Quantscript RT Kit (Tiangen, China) according to the manufacturer's instructions. Specific quantitative real‐time PCR (qRT‐PCR) assays were performed using PowerUp SYBR Green Master Mix (Applied Biosystems, USA) following the manufacturer's instructions. The primers used for qPCR are shown in Table S3, Supporting Information.

### RNA‐Immunoprecipitation

Cells were treated with or without ATRA (1 µg mL^−1^) for 72 h. The RIP assay was performed using the Magna RIP RNA‐binding protein immunoprecipitation kit (Millipore, USA) according to the manufacturer's instructions. Briefly, harvested cells were lysed with RIP lysis buffer, and immunoprecipitated with anti‐RAR*α* or control IgG antibodies at 4 °C overnight. The immunoprecipitated RNAs were purified and reverse transcribed to cDNA. Finally, the cDNAs were analyzed by end‐point RT‐PCR and 1.5% agarose gel electrophoresis.

### Dual‐Luciferase Assay

Both the 5’ UTR and 3’ UTR of OTUD6B were cloned into a luciferase reporter vector of pGL3‐Promoter, as shown in Figure 6M. The indicated plasmids and pRL‐TK were co‐transfected into HEK293T cells. After 24 h, the cells were treated with or without ATRA (1 µg mL^−1^). Finally, both firefly and *Renilla* luciferases were detected using the Dual‐luciferase Reporter Assay System (Promega, USA) according to the manufacturer's instructions. The luciferase activities were normalized to the *Renilla* luciferase activity of the internal control.

### RNA Sequencing

The total RNA was extracted with TRIzol reagent (Invitrogen, USA), and mRNA was isolated using magnetic beads with oligo‐dT. The cDNA was subjected to end‐repair and adaptor ligation using reverse transcriptase. The quality of the library was detected using an Agilent 2100 Bioanalyzer, and the concentration of the library was detect using Qubit 2.0. The RNAs were sequenced according to Illumina’ standard protocol. The RNA‐sequencing data are available at the Gene Expression Omnibus (GEO) database (accession GSE209521).

### Mass Spectrometry

Flag‐*β*‐TrCP or empty vector was transfected into HEK293T cells. After 24 h, the cells were treated with MG132 (20 µM) for 6 h. Harvested cells were lysed and incubated with anti‐Flag M2 affinity gel (A2220, Sigma Aldrich, USA) at 4 °C overnight. The immunoprecipitated proteins were resolved by gel electrophoresis, and the gel was silver‐stained using a Pierce Silver Stain Kit (Thermo Scientific, USA) according to the manufacturer's instructions. Finally, the bands were subjected to LC‐MS/MS analysis.

### Animal Studies

*Otud6b* conditional knockout mice (cKO, C57BL/6N background) were generated by crossing *Otud6b^LoxP/−^* mice with *ED‐L2‐Cre* mice, which were specifically expressed in the epithelia of the tongue, forestomach, ventral neck skin, and esophagus.^[^ ^29^ ^]^ The LoxP locus was located in the periphery of OTUD6B exon 2. The cKO mice were homozygous for floxed OTUD6B and hemizygous for the Cre transgene. For all experiments with *Otud6b* cKO mice, *Otud6b* WT mice served as the controls. 4NQO induction began at 6 weeks of age in the above male mice. The mice were treated with 4NQO in the drinking water at a concentration of 100 µg mL^−1^ for 16 weeks. After 4NQO treatment, the mice were given normal water until sacrifice. In the ATRA treatment group, the mice were given ATRA (5 mg kg^−1^) by gavage three times a week.

For the in vivo metastasis assay, 1 × 10^6^ cells were injected into the tail vein of 6‐week‐old male NOD/SCID mice purchased from Vital River (Beijing, China). The lung metastasis was imaged by the Xenogen in vivo imaging system. For the in vivo tumorigenic assay, various numbers (2 × 10^3^, 2 × 10^4^, 2 × 10^5^) of the indicated cells were subcutaneously injected into six‐week‐old BALB/C nude mice (Huafukang, Beijing). The tumors were harvested 6 weeks after injection. For in vivo ATRA therapeutic experiments, mice were given ATRA (5 mg kg^−1^) or corn oil by gavage three times a week. After 1 week, the indicated cells were injected subcutaneously into mice treated with ATRA or not and oral administration was continued. The tumors were harvested four weeks after injection.

For ATRA and mouse anti‐PD‐1 (anti‐mPD‐1) combination therapeutic experiment, 3 × 10^6^ cells were subcutaneously injected into 6‐week‐old C57BL/6N mice purchased from Vital River (Beijing, China). Mice were given ATRA and anti‐mPD‐1 antibody (BE0146, Bio X Cell) as shown in Figure 7H. Control group mice were given corn oil (gavage) and IgG2a isotype control (intraperitoneal injection, BE0089, Bio X Cell). ATRA group mice were given ATRA (5 mg kg^−1^) by gavage. Anti‐mPD‐1 group mice were given anti‐mPD‐1 (5 mg kg^−1^) by intraperitoneal injection. ATRA and anti‐mPD‐1 combination group mice were given ATRA (5 mg kg^−1^) by gavage and anti‐mPD‐1 (5 mg kg^−1^) by intraperitoneal injection.

All animal experiments were approved by the Institutional Animal Care and Use Committee of Chinese Academy of Medical Sciences Cancer Hospital. The animals were all randomized to different groups.

### Statistics

Unless otherwise specified, all results are shown as the mean ± SEM of independent experiments and were analyzed by unpaired *t*‐test. The correlation between OTUD6B and *β*‐TrCP or SNAIL in tissue specimens was calculated using Pearson's test. The expression of OTUD6B or *β*‐TrCP in ESCC tissues and normal tissues was analyzed by paired *t*‐test. Survival analysis was conducted using the Kaplan‐Meier model and analyzed by a two‐sided log‐rank test. For the in vivo tumorigenic assay, the tumor‐initiating frequency and statistics were analyzed by ELDA software.^[^ ^30^ ^]^ All other statistical analyses were performed with GraphPad Prism 8 software. A *p*‐value < 0.05 was considered statistically significant and denoted as * *p* < 0.05, ** *p* < 0.01, *** *p* < 0.001, and **** *p* < 0.0001 in all figures. NS, not significant (*p* > 0.05).

## Conflict of Interest

The authors declare no conflict of interest.

## Author Contributions

L.L., R.Z., and H.Z. designed the research and performed most experiments. X.Y., Y.L., and R.F. contributed to several experiments. L.L., Y.Y., and Y.L. performed data analysis. J.P.K., Y.Z., C.‐P.C., and L.Z. helped with the animal experiments. L.L. and Y.Z. wrote the manuscript. L.Z. and Z.L. supervised this project and revised the manuscript.

## Supporting information

Supporting Information

## Data Availability

The RNA‐seq data in this study has been deposited in the Gene Expression Omnibus with accession code GSE209521. PRJCA004501 dataset was accessed from BIG Data Center (http://bigd.big.ac.cn/gsa). The TCGA and GTEx data were accessed from UCSC Xena (http://xena.ucsc.edu/). The CPTAC data were accessed from UALCAN (http://ualcan.path.uab.edu/). GSE53622 dataset was accessed from NCBI GEO (https://www.ncbi.nlm.nih.gov/geo/).
