# Supplementary material for: All‐Trans Retinoic Acid Promotes a Tumor Suppressive OTUD6B‐β‐TrCP‐SNAIL Axis in Esophageal Squamous Cell Carcinoma and Enhances Immunotherapy
Source: Adv Sci (Weinh). 2023 Apr 10;10(16):2207458. doi: 10.1002/advs.202207458 (PMC10238178; doi:10.1002/advs.202207458)
Supplement: Supplementary file 1 — Supporting Information [file ADVS-10-2207458-s001.pdf]

ADVANCED SCIENCE
Open Access

Supporting Information

for *Adv. Sci.*, DOI 10.1002/advs.202207458

All-Trans Retinoic Acid Promotes a Tumor Suppressive OTUD6B-β-TrCP-SNAIL Axis in Esophageal Squamous Cell Carcinoma and Enhances Immunotherapy

Lei Li, Rui Zhu, Honghong Zhou, Chun-Ping Cui, Xiao Yu, Yuhao Liu, Yin Yin, Yang Li, Riyue Feng, Jonathan P. Katz, Yahui Zhao, Yun Zhang, Lingqiang Zhang* and Zhihua Liu**

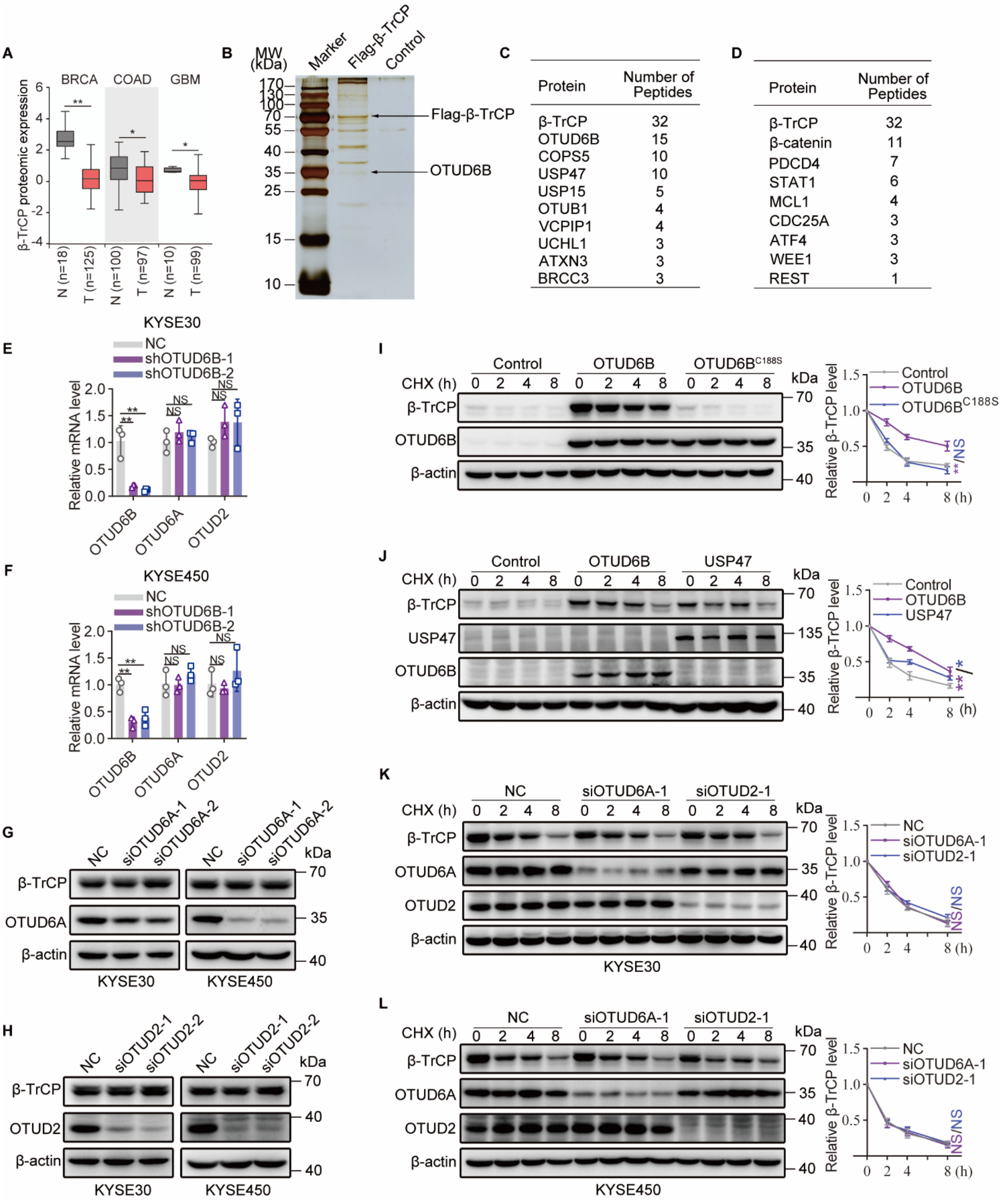
A
BRCA
COAD
GBM
β-TrCP proteomic expression
N (n=18)
T (n=125)
N (n=100)
T (n=97)
N (n=10)
T (n=99)
B
MW (kDa)
Marker
Flag-β-TrCP
Control
Flag-β-TrCP
OTUD6B
C
Protein | Number of Peptides
β-TrCP 32
OTUD6B 15
COPS5 10
USP47 10
USP15 5
OTUB1 4
VCPIP1 4
UCHL1 3
ATXN3 3
BRCC3 3
D
Protein | Number of Peptides
β-TrCP 32
β-catenin 11
PDCD4 7
STAT1 6
MCL1 4
CDC25A 3
ATF4 3
WEE1 3
REST 1
E
KYSE30
NC
shOTUD6B-1
shOTUD6B-2
Relative mRNA level
OTUD6B
OTUD6A
OTUD2
F
KYSE450
NC
shOTUD6B-1
shOTUD6B-2
Relative mRNA level
OTUD6B
OTUD6A
OTUD2
G
NC
siOTUD6A-1
siOTUD6A-2
β-TrCP
OTUD6A
β-actin
kDa
KYSE30
KYSE450
H
NC
siOTUD2-1
siOTUD2-2
β-TrCP
OTUD2
β-actin
kDa
KYSE30
KYSE450
I
Control
OTUD6B
OTUD6BC188S
CHX (h) 0 2 4 8
β-TrCP
OTUD6B
β-actin
kDa
Relative β-TrCP level
Control
OTUD6B
OTUD6BC188S
(h)
J
Control
OTUD6B
USP47
CHX (h) 0 2 4 8
β-TrCP
USP47
OTUD6B
β-actin
kDa
Relative β-TrCP level
Control
OTUD6B
USP47
(h)
K
NC
siOTUD6A-1
siOTUD2-1
CHX (h) 0 2 4 8
β-TrCP
OTUD6A
OTUD2
β-actin
kDa
KYSE30
Relative β-TrCP level
NC
siOTUD6A-1
siOTUD2-1
NS/NS
(h)
L
NC
siOTUD6A-1
siOTUD2-1
CHX (h) 0 2 4 8
β-TrCP
OTUD6A
OTUD2
β-actin
kDa
KYSE450
Relative β-TrCP level
NC
siOTUD6A-1
siOTUD2-1
NS/NS
(h)

Figure S1 OTUD6B enhances β-TrCP protein stability.

(A) The relative protein level of β-TrCP was analyzed using the proteomic profiles in CPTAC data (only BRCA, COAD and GBM subsets including the level of β-TrCP). mean ± SD, unpaired *t*-test, * $P < 0.05$, ** $P < 0.01$.

(B) The immunoprecipitated proteins of Flag-β-TrCP were resolved by gel electrophoresis and visualized by silver staining.

(C and D) The immunoprecipitated proteins of Flag-β-TrCP were detected by MS. The peptide numbers of DUB proteins associated with Flag-β-TrCP (C) and proteins known to bind with β-TrCP (D).

(E and F) The mRNA levels of OTUD6B, OTUD6A and OTUD2 in OTUD6B-knockdown KYSE30 (E) and KYSE450 (F) cells. n = 3, mean ± SEM, unpaired *t*-test, ** $P < 0.01$, NS, not significant ($P > 0.05$).

(G and H) The protein level of β-TrCP in OTUD6A-knockdown (G) or OTUD2-knockdown (H) KYSE30 and KYSE450 cells.

(I) OTUD6B or OTUD6B^{C188S} was transfected into HEK293T cells, and the cells were treated with CHX (50 μg/ml) for 2, 4, 8 hours. The degradation of β-TrCP was detected and quantified. n = 3, mean ± SD, unpaired *t*-test, ** $P < 0.01$, NS, not significant ($P > 0.05$).

(J) OTUD6B or USP47 was transfected into HEK293T cells, and the cells were treated with CHX (50 μg/ml) for 2, 4, 8 hours. The degradation of β-TrCP was detected and quantified. n = 3, mean ± SD, unpaired *t*-test, * $P < 0.05$, ** $P < 0.01$.

(K and L) OTUD6A-knockdown or OTUD2-knockdown KYSE30 (K) and KYSE450 (L) cells were treated with CHX (50 μg/ml) for 2, 4, 8 hours. The degradation of β-TrCP was detected and quantified. n = 3, mean ± SD, unpaired *t*-test, NS, not significant ($P > 0.05$).

The data shown in G-L are from a representative experiment in at least two replicates.

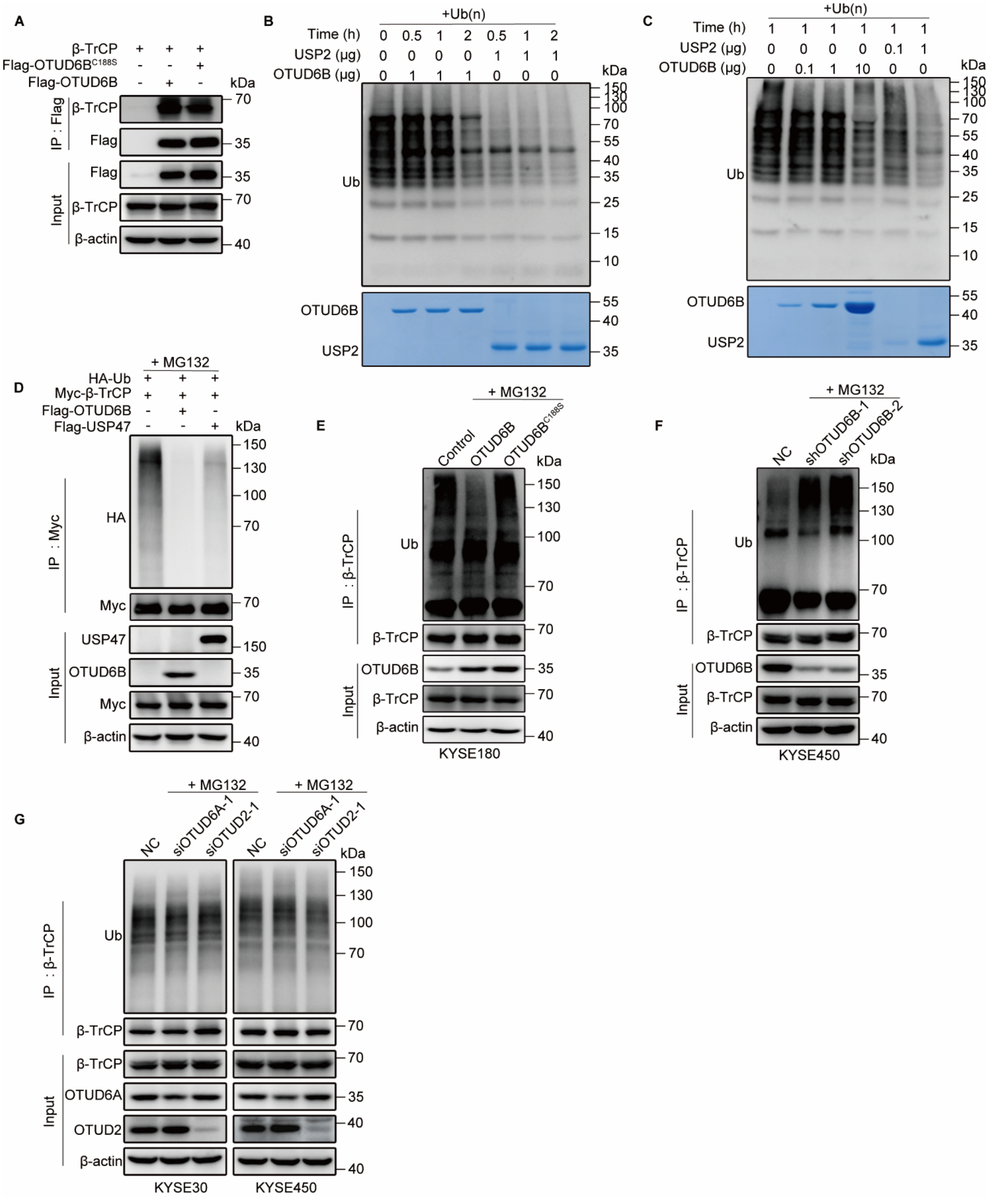

A
β-TrCP + + +
Flag-OTUD6B^C188S - - +
Flag-OTUD6B - + -
kDa
IP : Flag
β-TrCP
Flag
Input
Flag
β-TrCP
β-actin
70
35
35
70
40
B
+Ub(n)
Time (h) 0 0.5 1 2 0.5 1 2
USP2 (μg) 0 0 0 0 1 1 1
OTUD6B (μg) 0 1 1 1 0 0 0
kDa
Ub
OTUD6B
USP2
150
130
100
70
55
40
35
25
15
10
55
40
35
C
+Ub(n)
Time (h) 1 1 1 1 1 1
USP2 (μg) 0 0 0 0 0.1 1
OTUD6B (μg) 0 0.1 1 10 0 0
kDa
Ub
OTUD6B
USP2
150
130
100
70
55
40
35
25
15
10
55
40
35
D
+ MG132
HA-Ub + + +
Myc-β-TrCP + + +
Flag-OTUD6B - + -
Flag-USP47 - - +
kDa
IP : Myc
HA
Myc
Input
USP47
OTUD6B
Myc
β-actin
150
130
100
70
70
150
35
70
40
E
+ MG132
Control
OTUD6B
OTUD6B^C188S
kDa
IP : β-TrCP
Ub
β-TrCP
Input
OTUD6B
β-TrCP
β-actin
150
130
100
70
70
35
70
40
KYSE180
F
+ MG132
NC
shOTUD6B-1
shOTUD6B-2
kDa
IP : β-TrCP
Ub
β-TrCP
Input
OTUD6B
β-TrCP
β-actin
150
130
100
70
70
35
70
40
KYSE450
G
+ MG132
+ MG132
NC
siOTUD6A-1
siOTUD2-1
NC
siOTUD6A-1
siOTUD2-1
kDa
IP : β-TrCP
Ub
β-TrCP
Input
β-TrCP
OTUD6A
OTUD2
β-actin
150
130
100
70
70
70
35
40
40
KYSE30
KYSE450

Figure S2 OTUD6B directly interacts with and deubiquitinates β-TrCP.

(A) Flag-OTUD6B or Flag-OTUD6B^{C188S} plasmid was co-transfected with β-TrCP into HEK293T cells. The interaction of β-TrCP and OTUD6B or OTUD6B^{C188S} was detected by immunoprecipitation assay.

(B) The Ub(n)-ubiquitinylated substrate was incubated with purified GST-OTUD6B or USP2 for 0.5, 1, 2 hours at 37 °C. USP2 was used as a positive control. The status of ubiquitin was analyzed by immunoblotting.

(C) The Ub(n)-ubiquitinylated substrate was incubated with purified GST-OTUD6B (0.1, 1, 10 μg) or USP2 (0.1, 1 μg) for one hour at 37 °C. The status of ubiquitin was analyzed by immunoblotting.

(D) Myc-β-TrCP and HA-Ub were co-expressed with Flag-OTUD6B or Flag-USP47 into HEK293T cells. The ubiquitination level of β-TrCP was detected using an anti-HA antibody.

(E and F) The ubiquitination level of β-TrCP in OTUD6B- or OTUD6B^{C188S}- overexpressing KYSE180 cells (E) and OTUD6B-knockdown KYSE450 cells (F).

(G) The ubiquitination level of β-TrCP in OTUD6A-knockdown or OTUD2-knockdown KYSE30 and KYSE450 cells.

All data from a representative experiment in at least two replicates.

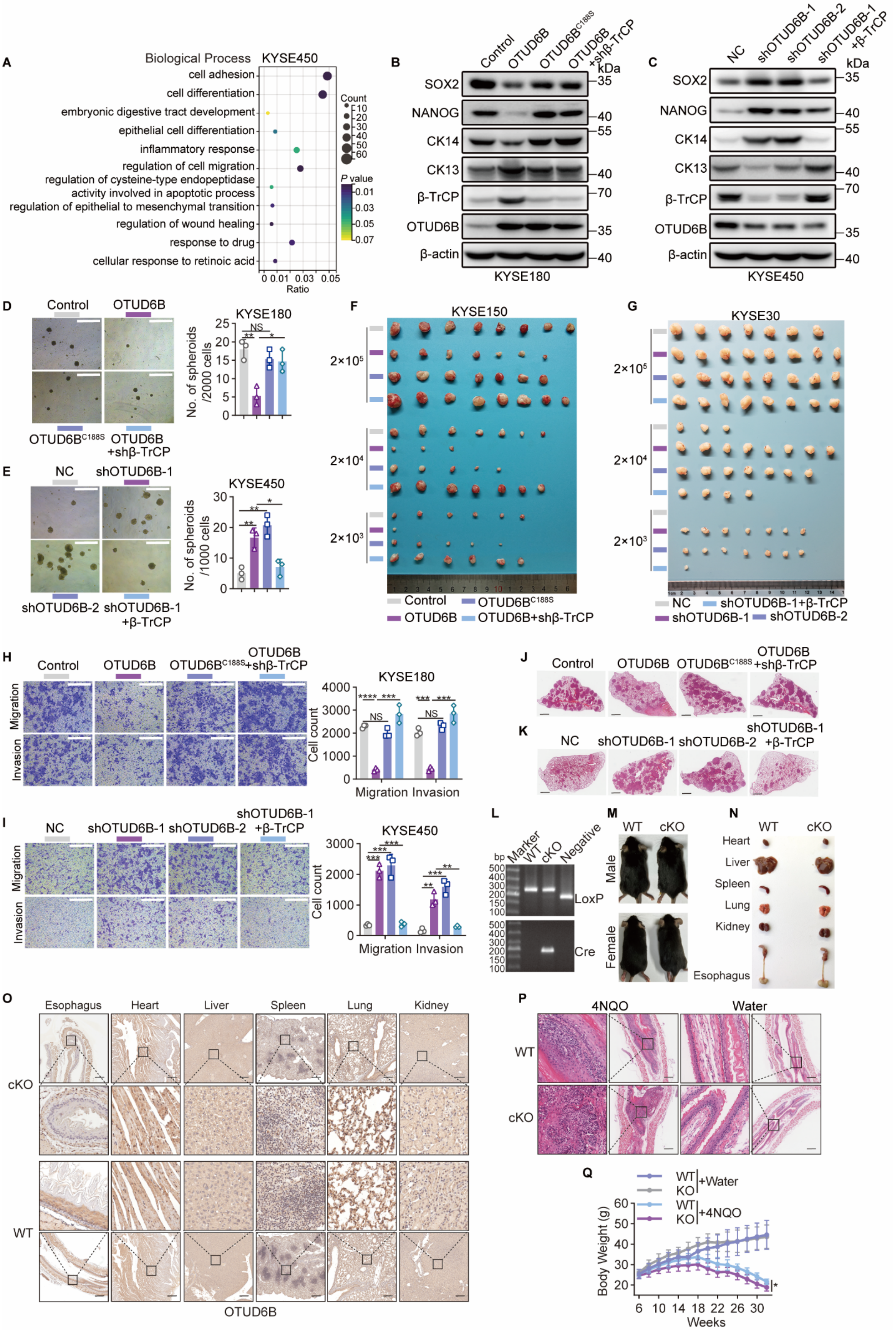

A
Biological Process KYSE450
cell adhesion
cell differentiation
embryonic digestive tract development
epithelial cell differentiation
inflammatory response
regulation of cell migration
regulation of cysteine-type endopeptidase activity involved in apoptotic process
regulation of epithelial to mesenchymal transition
regulation of wound healing
response to drug
cellular response to retinoic acid
Ratio
Count
P value
B
Control
OTUD6B
OTUD6B C188S
OTUD6B +shβ-TrCP
kDa
SOX2
NANOG
CK14
CK13
β-TrCP
OTUD6B
β-actin
KYSE180
C
NC
shOTUD6B-1
shOTUD6B-2
shOTUD6B-1 +β-TrCP
KYSE450
D
KYSE180
No. of spheroids /2000 cells
E
KYSE450
No. of spheroids /1000 cells
F
KYSE150
G
KYSE30
H
Migration
Invasion
Cell count
I
J
K
L
Marker
WT
cKO
Negative
bp
LoxP
Cre
M
Male
Female
N
Heart
Liver
Spleen
Lung
Kidney
Esophagus
O
Esophagus
Heart
Liver
Spleen
Lung
Kidney
cKO
WT
OTUD6B
P
4NQO
Water
Q
WT
KO
+Water
+4NQO
Body Weight (g)
Weeks

Figure S3 OTUD6B reduces TIC properties of ESCC cells *via* β-TrCP.

(A) KYSE450 cells with or without OTUD6B-knockdown were subjected to RNA-seq and gene ontology (GO) analysis. A bubble chart shows the partial enriched terms of biological processes. The RNA-sequencing data are available at the GEO database (accession GSE209521).

(B and C) The levels of SOX2, NANOG, CK14 and CK13 in the indicated KYSE180 cells (B) and KYSE450 cells (C). Data from a representative experiment in at least two replicates.

(D and E) The spheroid formation capacity of the indicated KYSE180 cells (D) and KYSE450 cells (E) were detected by the spheroid formation assay. Scale bars, 500 μm. n = 3, mean ± SEM, unpaired *t*-test, * $P < 0.05$, ** $P < 0.01$, NS, not significant ($P > 0.05$).

(F and G) The indicated KYSE150 (F) and KYSE30 (G) cells were subcutaneously injected into BALB/c nude mice to detect the tumor formation rates.

(H and I) The migration and invasion capabilities of the indicated KYSE180 cells (H) and KYSE450 cells (I). Scale bars, 500 μm. n = 3, mean ± SEM, unpaired *t*-test, ** $P < 0.01$, *** $P < 0.001$, **** $P < 0.0001$, NS, not significant ($P > 0.05$).

(J and K) The indicated KYSE150 cells (J) and KYSE30 cells (K) were injected into the tail vein of NOD/SCID mice, and the H&E staining of indicated lungs. Scale bars, 2 mm.

(L) The genotype of *Otud6b* WT and cKO mice tail.

(M) Representative images of *Otud6b* WT and cKO mice.

(N) Representative images of organs of *Otud6b* WT and cKO mice.

(O) IHC staining of OTUD6B in the indicated organs of *Otud6b* WT and cKO mice.

(P) H&E staining of the esophagus in 4NQO-induced *Otud6b* WT and cKO mice.

(Q) The body weights of 4NQO-induced *Otud6b* WT and cKO mice. mean ± SD, unpaired *t*-test, * $P < 0.05$.

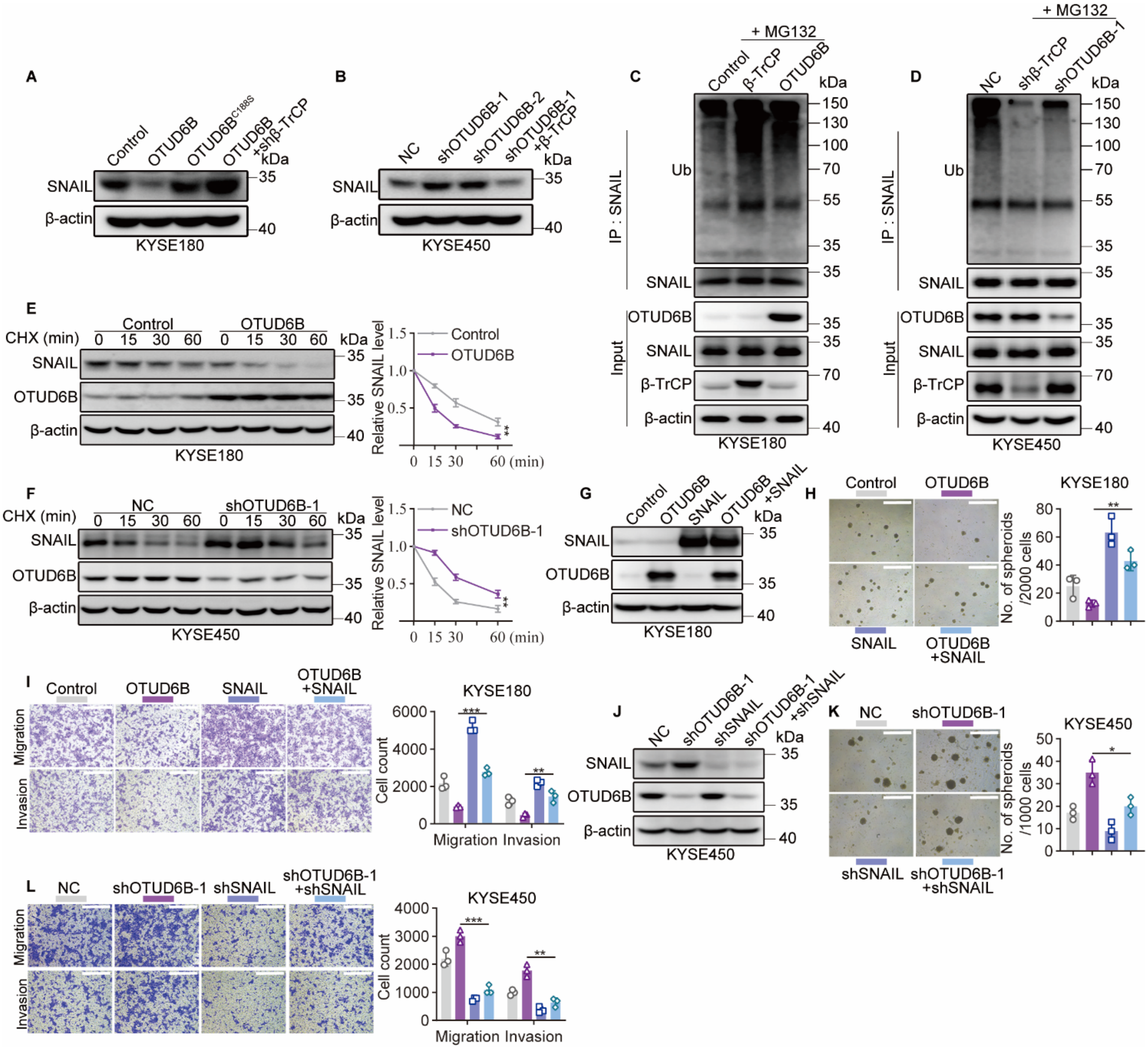

Figure S4 OTUD6B promotes the degradation of SNAIL.

(A and B) The levels of SNAIL in the indicated KYSE180 cells (A) and KYSE450 cells (B) were detected by immunoblotting.

(C) The ubiquitination level of SNAIL in OTUD6B- or β-TrCP- overexpressing KYSE180 cells.

(D) The ubiquitination level of SNAIL in β-TrCP-knockdown or OTUD6B-knockdown KYSE450 cells.

(E and F) The degradation of SNAIL in OTUD6B-overexpressing KYSE180 cells (E) or OTUD6B-knockdown KYSE450 cells (F) treated with CHX (50 μg/ml) for 15, 30, 60 minutes. n = 3, mean ± SD, unpaired *t*-test, ** $P < 0.01$.

(G) SNAIL was overexpressed in OTUD6B-overexpressing or control KYSE180 cells. The levels of

SNAIL and OTUD6B were detected by immunoblotting.

(H and I) The spheroid formation capacity (H) and migration and invasion capabilities (I) of the indicated KYSE180 cells. Scale bars, 500 μm. n = 3, mean ± SEM, unpaired *t*-test, ** $P < 0.01$, *** $P < 0.001$.

(J) SNAIL was knocked down in OTUD6B-knockdown KYSE450 cells. The levels of SNAIL and OTUD6B were detected by immunoblotting.

(K and L) The spheroid formation capacity (K) and migration and invasion capabilities (L) of the indicated KYSE450 cells. Scale bars, 500 μm. n = 3, mean ± SEM, unpaired *t*-test, * $P < 0.05$, ** $P < 0.01$, *** $P < 0.001$.

The data shown in A-F are from a representative experiment in at least two replicates.

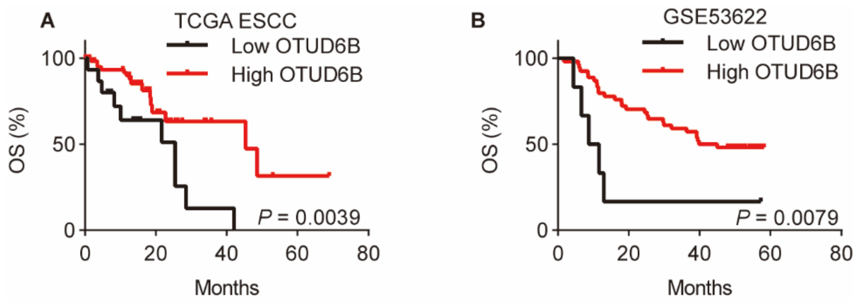

Figure S5 Reduced OTUD6B predicts a poor prognosis in ESCC patients.

(A and B) Kaplan-Meier plot of the overall survival of ESCC patients stratified by OTUD6B expression using the TCGA ESCC (A) and GSE53622 (B) datasets. Two-sided log-rank test.

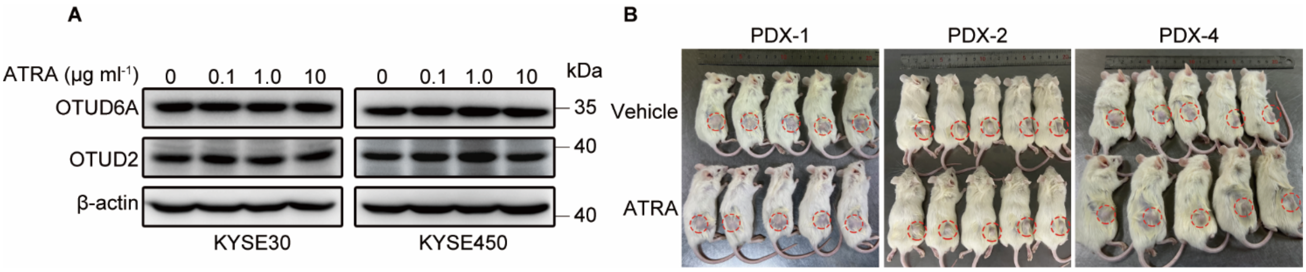

Figure S6 OTUD6B is critical for ATRA-mediated inhibition of TIC properties in ESCC.

(A) The protein levels of OTUD6A and OTUD2 in KYSE30 and KYSE450 cells treated with ATRA (0.1, 1, 10 μg/ml). Data from a representative experiment in at least two replicates.

(B) Images of PDX-1, PDX-2 and PDX-4 mice treated with or without ATRA (5 mg/kg).

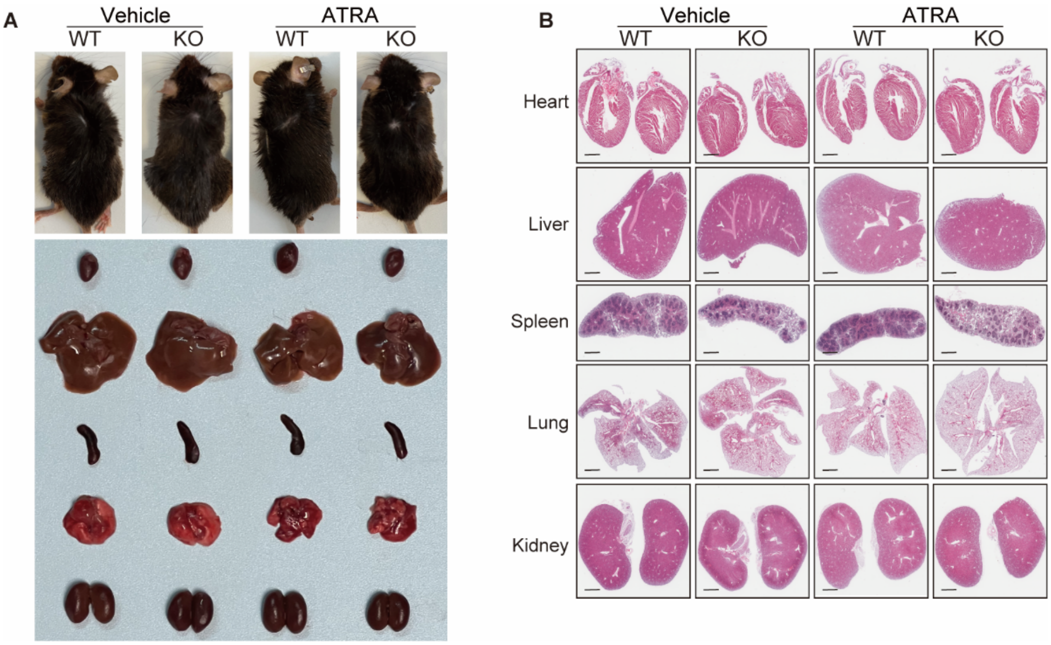

Figure S7 ATRA enhances the response of established ESCC tumors to anti-PD-1 immunotherapy.

(A and B) Representative images (A) and H&E staining (B) of the indicated organs in 4NQO-induced *Otud6b* WT and cKO mice treated with or without ATRA.

Table S1. List of primers used for plasmids construction and target sequence of shRNAs and siRNAs.

Genes	Forward Primer 5'-3'	Reverse Primer 5'-3'
OTUD6B	TTGGATCCGCCACCATGGAGG CGGTATTGACCGAAGAGC	TTGCGGCCGCTTAGCTGCAAT TTTCAGTAACTATG
OTUD6B^{C188S}	AGATTCCATCTGATGGCCACAG TATGTATAAAGCCA	TTCAATGGCTTTATACATACTG TGGCCATCAGATGG
GST-OTUD6B-N	TTGGATCCGCCACCATGGAGGC GGTATTGACCGAAGAGC	TTGCGGCCGCTTAAATCTGTTT AATTTCTAACTGT
GST-OTUD6B-C	TTGGATCCGCCACCATGCCATC TGATGGCCACTGTATGT	TTGCGGCCGCTTAGCTGCAATT TTCAGTAACTATG
OTUD6B 5'UTR	AAGCTTGCGCACGCGCAGCAC CCCATTTAAG	CCATGGTGGCTACGGCTGGGA CCCAGCCCCG
OTUD6B 3'UTR	GGATCCTCTAGATTTATACAAT GTTGTACAATTATGT	GCGGCCGCTCTAGATGTTGATT TAATTCATTCATTTTTT
β-TrCP	TTGGATCCGCCACCATGGACCC GGCCGAGGCGGTGCTGC	TTACTAGTTTATCTGGAGATGT AGGTGTATGTT
GST-β-TrCP-N	TTGGATCCGCCACCATGGACCC GGCCGAGGCGGTGCTGC	TTGCGGCCGCTTAACTTCGGCA GTGAATTCTCTGT
GST-β-TrCP-C	TTGGATCCGCCACCATGGAAAC AAGCAAAGGAGTTTACT	TTACTAGTTTATCTGGAGATGT AGGTGTATGTT
SNAIL	TTGGATCCGCCACCATGCCGCG CTCTTTCCTC	TTGCGGCCGCGCGGGGACATC CTGAGCAGCCGGAC
RARα	TTGGATCCGCCACCATGGCCAG CAACAGCAGCTCCTGCC	TTACTAGTTCACGGGGAGTGGG TGGCCGGGCTG
ATXN3	TTGGATCCGCCACCATGGAGTC CATCTTCCACGAGAAAC	TTCCGCGGTTTTTTTCCTTCTGT TTTCAAATCA
BRCC3	TTGCTAGCGCCACCATGGCGGT GCAGGTGGTGCAGGCGG	TTCCGCGGTTCTAGAGAAGAA AGTTCTTGCATA
COPS5	TTGGATCCGCCACCATGGCGGC GTCCGGGAGCGGTATGG	TTCCGCGGAGAGATGTTAATTT GATTAAACAGT
USP15	TTGCTAGCGCCACCATGGCGGA AGGCGGAGCGGCGGATC	TTCCGCGGGTTAGTGTGCATAC AGTTTTCATTT
USP47	TTGCTAGCGCCACCATGGTGCC CGGCGAGGAGAACCAAC	TTCCGCGGGTCTTGAGTCAGAT CTTTATTTGGT
UCHL1	TTGGATCCGCCACCATGCAGCT CAAGCCGATGGAGATCA	TTCCGCGGGGCTGCCTTGCAGA GAGCCACGGCA
OTUB1	TTGCTAGCGCCACCATGGCGGC GGAGGAACCTCAGCAGC	TTTCTAGATTTGTAGAGGATAT CGTAGTGTCCA
VCPIP1	TTGCTAGCGCCACCATGTCTCA GCCGCCGCCGCCGCCGC	TTCCGCGGAGAGTGATCCATTG GCTCAGTTGTG
shOTUD6B-1	CAATTGAAGCTGACTACTA	/
shOTUD6B-2	CTAGACAGTTAGAAATTAA	/

shβ-TrCP	CTGGAGGCAGATGACATCTAA	/
shSNAIL	CCACTCAGATGTCAAGAAGTA	/
siRARα-1	GTGAGAAACGACCGAAACA	/
siRARα-2	CTCAGAACAACGTGTCTCT	/
siOTUD6A-1	GCACTACAACTCCGTGACA	/
siOTUD6A-2	CCAGCTACATGAAGAAGCA	/
siOTUD2-1	GGGATACCATTCTGGAAGA	/
siOTUD2-2	GGAGCAATAGAGATATCGA	/

Table S2. List of primary antibodies

Name	Cat#	Company	Application
OTUD6B	NBP1-85652	Novus Biologicals	IB: 1:500, IF: 1:100, IHC: 1:100
OTUD6B	PA5-110067	Invitrogen	IP: 1:100
β-TrCP	#4394	Cell Signaling Technology	IB: 1000, IP: 1:100
β-TrCP	37-3400	Invitrogen	IF: 1:200
β-TrCP	ab71753	Abcam	IHC: 1:100
SNAIL	#3879	Cell Signaling Technology	IB: 1000
SNAIL	sc-28199	Santa Cruz Biotechnology	IF: 1:100, IP: 1:100
SNAIL	ab180714	Abcam	IHC: 1:100
Ubiquitin	#3936	Cell Signaling Technology	IB: 1000
SOX2	#3579	Cell Signaling Technology	IB: 1000
SOX2	ab92494	Abcam	IHC: 1:100
NANOG	#4903	Cell Signaling Technology	IB: 1000
NANOG	ab214549	Abcam	IHC: 1:100
CK13	10164-2-AP	Proteintech	IB: 1:5000, IHC: 1:100
CK14	60320-1-lg	Proteintech	IB: 1:1000
RARα	#62294	Cell Signaling Technology	IB: 1000, RIP: 100
GST	#2625	Cell Signaling Technology	IB: 1000
HA	#3724	Cell Signaling Technology	IB: 1000
Flag	#14793	Cell Signaling Technology	IB: 1000
Myc-tag	562	MBL	IB: 2000
β-actin	A5316	Sigma Aldrich	IB: 5000
OTUD6A	24486-1-AP	Proteintech	IB: 1000
OTUD2	25370-1-AP	Proteintech	IB: 1000
Mouse PD-1	BE0146	Bio X Cell	5 mg/kg
IgG2a control	BE0089	Bio X Cell	5 mg/kg

immunoblotting (IB), immunofluorescence (IF), immunoprecipitation (IP), immunohistochemistry (IHC), RNA-immunoprecipitation (RIP).

Table S3. List of primers used for qPCR

Genes	Forward Primer 5'-3'	Reverse Primer 5'-3'
OTUD6B	AAGAGACGGGAAAAGAAAGC	ATTAGGGTTTGTTAAAAATGGCAGA
β-TrCP	ACCAACATGGGCACATAAACTC	TGGCATCCAGGTATGACAGAAT
OTUD6A	ATGGATGATCCGAAGAGTGAACA	GGTCTTGGGGACCGAGTTTT
OTUD2	GGTCAGCGAATCCTCGTCG	CACCACGTTTAGTAAATGCAGGT
GAPDH	AGGGCTGCTTTTAACTCTG	CTGGAAGATGGTGATGGG
